# A community-engaged investigation of residential polycyclic aromatic hydrocarbon exposures in West Eugene, OR

**DOI:** 10.1038/s41370-026-00863-w

**Published:** 2026-04-08

**Authors:** Francesca Germano, Lane G. Tidwell, Duo Jiang, Arjorie Arberry-Baribeault, Lisa Arkin, Michael Barton, Kim A. Anderson, Diana Rohlman

**Affiliations:** 1 College of Health, Oregon State University, Corvallis, 97331 OR USA; 2 Department of Environmental and Molecular Toxicology, Oregon State University, Corvallis, 97331 OR USA; 3 Department of Statistics, Oregon State University, Corvallis, 97331 OR USA; 4 Beyond Toxics, Eugene, 97440 OR USA

**Keywords:** Polycyclic aromatic hydrocarbons, Creosote, Community-engaged research, Passive sampling devices, Silicone wristbands, Report-back of research results

## Abstract

**Background:**

A West Eugene, OR community has a history of odor complaints, anecdotally linked to a nearby wood preservative facility using creosote, a known source of polycyclic aromatic hydrocarbons (PAHs). The community also experiences elevated cancer risks.

**Objective:**

In response to concerns about industrial air pollution, Beyond Toxics (BT) and Oregon State University (OSU) initiated a community-engaged study to characterize residential PAH exposure.

**Methods.:**

Stationary passive samplers were deployed in residential and commercial areas at 17 locations in three rings around the facility: inner (0.25-mile, *n* = 4), middle (0.5-mile, *n* = 5), and outer (1 mile, *n* = 8), for seven days. Twelve residents also wore personal passive wristband samplers (WBs), with eight hosting both a wristband and stationary sampler. All samplers were analyzed for 64 PAHs. Daily activity logs were collected to assess co-variate exposures. Results were shared through individual and community reports and in-person meetings.

**Results:**

Thirty-eight PAHs were detected in stationary samplers. The five most abundant were naphthalene (169 ng/m³), acenaphthene (165 ng/m³), 2-methylnaphthalene (160 ng/m³), 1-methylnaphthalene (87.0 ng/m³), and fluorene (40.4 ng/m³). Seventeen PAHs were detected across the 12 wristbands, with phenanthrene, 2-methylnaphthalene, acenaphthene, fluorene, and naphthalene as the most abundant. PAHs were highest in the inner ring and northeastern area, downwind of the facility, followed by the east, near an industrial railway.

**Significance:**

The exposure patterns observed reflect community reports of odors in the northeast. The most abundant PAHs in both sampler types are associated with creosote. All wristband PAHs were also observed in stationary samplers, suggesting a common exposure source. This community-engaged study identified higher exposures near the industrial source in both ambient and personal samples- supporting long-standing community concerns.

**Impact statement:**

Residents in an environmental justice community raised concerns about air pollution from industrial sources. A community-engaged research study used passive samplers to characterize and quantify ambient and personal exposure to vapor phase polycyclic aromatic hydrocarbons.

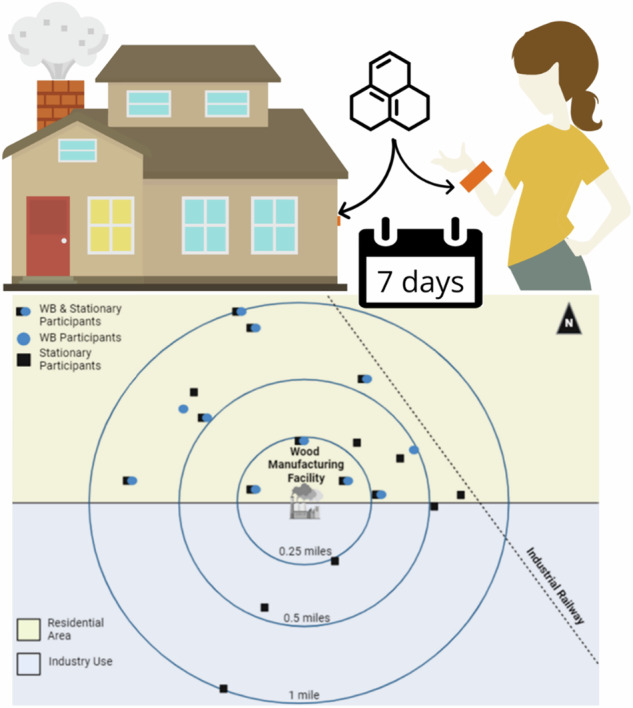

## Introduction

West Eugene, Oregon is an environmental justice community that borders many sources of environmental pollution. Residents live within a quarter mile of an industrial wood manufacturing facility and multiple other industrial sources, including railways. Since 1977, residents have registered complaints with the Lane Regional Air Protection Agency (LRAPA) regarding odors from the wood manufacturing facility [1].

The area has a strong history of community organizing and advocacy, led by the non-profit Beyond Toxics (BT), and others. Focused on representing environmental health concerns, BT partners with communities, researchers, and agencies to generate research data in collaboration with the community. In West Eugene, community concerns centered around air quality, given the use of creosote at the facility, and history of noxious odors. In 2005, the local health authority conducted air sampling (1–24-h sampling duration) for polycyclic aromatic hydrocarbons (PAHs), a common air pollutant, at 12 locations within West Eugene [1]. The locations were predominantly downwind (North) of the industrial wood processing facility in residential areas, as well as in the south (zoned for industrial use). However, reports by BT and LRAPA suggest residents to the northeast are similarly impacted by odors [1]. The previous sampling was conducted under conditions that were expected to result in maximum exposure: locations were predominantly located within one-quarter mile of the facility, during maximum industrial activity. While those results indicated levels of naphthalene were not of concern to human health [1], residents continued to report odor violations and concerns regarding their health.

In 2020, given the continued concerns, BT and researchers at Oregon State University (OSU) co-developed a community-engaged research study to assess exposure to PAHs. The study was heavily informed by the prior air monitoring in 2005, and by research characterizing PAH emissions from creosote-treated wood [2]. PAH emissions can be found, for example, from creosote-treated wood that is stored at a facility to dry [2]. Specifically, studies on PAHs released in coal tar creosote vapor have identified naphthalene, phenanthrene, fluoranthene and pyrene [2]. Thus, there is the potential for individuals living near wood-preservative plants to be exposed; a study in Delson, California found that urinary metabolites for naphthalene were higher in individuals living under 0.25 miles downwind of a facility using creosote [3].

PAHs are organic environmental contaminants derived from organic materials like petroleum and coal, and are made of two or more fused benzene rings [4]. Additionally, a significant source of PAHs in the environment is incomplete combustion [4]. PAHs are ubiquitous in the environment, either in the gaseous state, or by adsorbing onto particulate matter [5]. While both particulate and vapor phase PAHs have been evaluated for impacts on human health, there are gaps in scientific literature surrounding exclusively vapor phase PAHs, despite increased carcinogenic and mutagenic potential [6]. Vapor phase PAHs have a higher vapor pressure than those in the particulate phase, and can contribute to indoor PAH exposures due to their ability to enter buildings through open windows, doors, etc [7]. Vapor phase PAHs are also known to have higher emission factors than particulate phase PAHs, potentially increasing exposure [8].

Following conversations with BT and the community, the concern over exposure to PAHs as a function of proximity to the facility was identified, as well as the types and amounts of PAHs the community might be exposed to, and how those exposures compared to other US communities. To assess ambient and personal exposure to PAHs, stationary and personal passive samplers were utilized. Passive samplers are common tools used to detect ambient air pollutants such as PAHs in a low-cost, effective manner [9]. Working with BT and the community, samplers were deployed for seven days, and the results were reported back to study participants and the community.

## Materials and methods

### Site description

Located in West Eugene, an industrial wood preservative facility is situated within a mix of residential and industrial areas. The 31-acre facility, operating since the early 1940s [1], has been the subject of odor complaints since 1977. Around the site, soil and groundwater are contaminated with arsenic, pentachlorophenol, dioxin, and PAHs [1]. Residential areas are to the west, north and east, with industrial-use parcels to the south, and bordered by a railroad to the east. Sampling occurred from Oct. 11-18, 2021. As of January 31, 2022, the facility is no longer active [10]. On September 4, 2024, the site was nominated to be added to the Superfund National Priorities List [11].

### Study population, recruitment methodology, and training

All study activities were reviewed and approved by the Institutional Review Board at Oregon State University (IRB-2021-1087). Beyond Toxics staff conducted all recruitment activities. A mix of virtual outreach events, dissemination through existing list-servs, social media, flyers, and email was used to raise awareness about the study and conduct recruitment. Interested participants set up a phone call with study staff to affirm interest in the study, determine their eligibility (age 18 or older or participating with consent of legal guardian for an individual under the age of 18, speak English or Spanish, live within 1 mile radius of the facility), and assess their interest in wearing a wristband, hosting an air sampler on their property, or both. Participants were reminded of the study activities (seven-day participation, fill out surveys, record daily activities, wear a wristband and/or use a stationary sampler). Eligible and willing participants were then sent a link to a Qualtrics® (Qualtrics, Provo, UT) questionnaire to collect informed consent as well as socio-demographic information (Table 1). Upon providing informed consent, participants were contacted by phone or email to schedule the start of the study.

**Table 1:** Demographics of study participants.

	Survey Results			
	Wristband Participants only *n* (%)	Stationary Participants only *n* (%)	WB and Stationary Participants *n* (%)	Total Participants *n* (%)
**Age**				
18–35 years of age	3 (60%)	0	1 (20%)	4 (23.5%)
36–65 years of age	2 (40%)	5 (71.4%)	4 (80%)	11 (64.7%)
66 years of age or older	0	2 (28.6%)	0	2 (11.7%)
**Race/Ethnicity**				
White	5 (100%)	7 (100%)	5 (100%)	17 (100%)
**Gender**				
Male	2 (40%)	0	2 (40%)	4 (23.5%)
Female	3 (60%)	7 (100%)	3 (60%)	13 (76.5%)
**Income Level**				
$10,000-$39,999	0	2 (57.1%)	4 (57.1%)	6 (35.3%)
$50,000-$79,999	0	1 (14.3%)	3 (42.9%)	4 (23.5%)
$80,000 or more	3 (40%)	0	0	3 (17.6%)
Prefer not to answer	1 (20%)	3 (42.9%)	0	4 (23.5%)
**Education Level**				
High school	1 (20%)	1(14.3%)	1 (14.3%)	3 (17.6%)
Some college	0	4 (57.1%)	2 (28.6%)	6 (35.3%)
Associate degree	1 (20%)	1(14.3%)	0	2 (11.8%)
Bachelor’s degree	2 (40%)	1(14.3%)	2 (28.6%)	5 (29.4%)
Master’s degree	1(20%)	0	1 (13.3%)	1 (5.9%)
Prefer not to answer	0	0	0	0
**Smoking Status**				
Yes	0	2 (40%)	4 (57.1%)	6 (35.3%)
No	5 (100%)	3 (60%)	3 (42.9%)	11 (64.7%)

Sampling was conducted from October 11–18^th^, 2021. Given the ongoing COVID-19 pandemic, in-person community participation and engagement were deliberately limited. Research staff wore masks and provided masks to any study participants as a courtesy. Wristband kits (Fig. S1) were personalized to each participant and left on their doorstep. For stationary passive samplers, participants either provided written permission (email) for research staff to enter their property and set up the sampler or met outside with the study team at a distance to oversee the selection of sampler location. Information about the stationary samplers was also provided (Fig. S1).

Upon completion of the study, participants placed the wristband back into the study kit in a sealed, airtight Teflon® bag, which they left on their front porch for retrieval. Written permission was again obtained to enter properties to retrieve the stationary samplers. Participants received a gift card ($50) per sampler. All participants had the option during consent to receive the results from their sampler(s) and 100% requested their results.

### Exposure assessment

#### Determination of sampling locations

Concentric sampling rings were visualized around the facility (Fig. S2), based on prior research showing emissions from coal tar creosote are highest within 0.25 miles, and significantly decrease by 1.0 miles [3]. Thus, the sampling rings comprised an inner ring (0–0.25-mile), middle ring (0.25–0.5-mile) and outer ring (0.5–1-mile). Locations were selected based on alignment with a sampling ring, and to represent the four cardinal directions plus the NE and NW given the history of odor complaints.

#### Stationary samplers

Stationary samplers were deployed at 17 locations around the facility (Fig. S2; inner ring= 4, middle ring= 4, outer ring= 9). One location was deployed in triplicate as part of our data quality objectives. Samplers were deployed for seven days. An example of a deployed sampler is shown in Fig. S3. Stationary samplers were submitted for analysis at the Food Safety and Environmental Stewardship (FSES) laboratory at Oregon State University.

#### Wristband samplers

13 participants wore passive sampling WBs during the same seven-day period. One participant lost their WB. The remaining 12 WBs were submitted for analysis at FSES.

#### Wristband compliance

Participants were instructed to complete labeling on their WB kit that reported their WB ID number and on/off times (Fig. S1) that were later used by the research team to ensure compliance. Upon return, WBs were assessed to ensure compliance with the study protocol: (i) airtight seal on the stored Teflon® bag, and (ii) worn for seven days ( ± 24 h). All 12 returned WBs met compliance criteria and were analyzed for 64 PAHs.

### Chemical exposure assessment

#### Stationary sampler preparation and extraction

Stationary samplers consist of a light-weight metal box (Fig. S3) containing five strips of low-density polyethylene (LDPE). LDPE was conditioned for field deployment using hexanes as described previously [12]. Each LDPE strip was infused with performance reference compounds (PRCs) to enable calculation of in situ sampling rates and time-integrated air concentrations [13]. Here, the PRCs were fluorene-d10, pyrene-d10 and benzo[b]fluoranthene-d12. Further information on air concentration calculations can be found in the calculations section of the Supplementary Information document. Samplers were cleaned after deployment in two isopropanol baths, stored in amber jars at -20°C. Prior to extraction, samples were spiked with deuterated PAHs to act as surrogates, allowing for quantification of extraction efficiency. LDPE was extracted in two rounds n-hexane, extracts were combined and concentrated to 1 mL and stored in amber glass gas chromatography vials at -20°C.

#### Silicone-wristband preparation and extraction

Wristbands were purchased from 24hourwristbands.com (Houston Texas). Wristbands were conditioned by rinsing each WB in deionized water, followed by baking in a vacuum oven (Blue-M POM18VC paired with welch duo seal pump model 1405) to remove particulate matter and compounds that result in analytical interference [14]. Conditioned samplers were stored at 4°C in airtight containers until deployment [14]. Additional details can be found in the seminal publication describing their use, and further details on the preparation and performance have been published. All wristbands were of the same size and weight [15]. Wristband passive samplers used in this investigation had an average mass of 4.5 grams, a circumference of 20 cm and are 1.5 mm in thickness [15].

#### PAH screening

LDPE and wristband extracts were quantitatively analyzed for 64 PAHs using an Agilent 7890 A gas chromatograph interfaced with an Agilent 7000 GC/MS-MS, as described previously [16]. Briefly, an Agilent Select PAH column was used and each PAH was calibrated with a curve of at least five points, with correlations ≥0.99. Limits of detection (LODs) range from 0.24 to 1.7 ng/mL, and limits of quantitation (LOQs) range from 1.0 to 7.1 ng/mL, except for two compounds that have higher LODs and LOQs. A list of PAHs, LODs and LOQs in the instrument method is included in Table S1. Background subtraction was performed using a trip blank.

#### Identification of PAHs found in creosote

Creosote is known to contain PAHs [17]. The PAHs in the analytic method were cross-referenced with PAHs known to be included in the contents or byproducts of industrial coal-tar creosote use. These ‘creosote-relevant’ PAHs were identified by government or peer-reviewed literature and are described in Table S2. Although the identified PAHs are commonly associated with creosote and were selected based on their relevance to known constituents and byproducts of industrial creosote use, it is important to acknowledge that these compounds are not exclusive to creosote. However, given the context of this study and the historical use of creosote at the site under investigation, these ‘creosote-relevant’ PAHs had been prioritized for analysis. We recognize that some portion of the measured PAHs may derive from other environmental sources, but their inclusion is based on their documented presence in creosote and relevance to the specific industrial activities at this location.

### Exposure survey and daily activity log

A Qualtrics® questionnaire was developed to capture informed consent (WB participants only), socio-demographic information, household characteristics (age of home, use of heating/cooling) and household product usage (e.g., pesticide applications, smoking indoors, air fresheners), and the option for participants to receive their study results. The survey was conducted using Qualtrics^xm^ software (Seattle, WA, USA), and consisted of 38 questions (Table S3).

All participants received an email with a link to complete the questionnaire. Using embedded logic, the questionnaire was personalized to the level of involvement (e.g., wearing a wristband, using a stationary monitor, completing both activities). For example, individuals wanting to participate in the wristband portion of the research were shown a mandatory informed consent section prior to beginning the survey. All wristband participants completed a questionnaire; participants with only a stationary monitor were requested to complete the questionnaire, and 95% did so. Data was extracted from Qualtrics and exported for further analysis to Microsoft Excel.

Daily activity logs were provided to account for any significant exposure to known PAH co-variates. Co-variates accounted for included burning candles or incense, smoking, burning food, opening windows, and other exposures to industrial products. Participants were asked to fill out the activity log every day of the study.

Activity logs were only included for analysis if at least five of the seven days included information; five participants left the packet blank. For both the activity logs and survey responses, only questions with a 40% response rate were evaluated (Table 2), and only questions with a 40% affirmative response were included for statistical analysis. Only five questions met these criteria from the daily activity log, and one question from the questionnaire (Table 2).

**Table 2:** Daily activity log and survey covariate responses.

Co-Variate	*N*, % (Stationary)	*N*, % (WB)	*P*-value (Stationary)	*P*-value (WB)
*Electric Heat Duration*				
0-60 h 60-120 h 120+ h	3 (42.9%) 1 (13.3%) 3 (42.9%)	7 (63.6%) 1 (9.1%) 3 (27.3%)	- - -	- - -
*AC/Filtration Duration*				
0-60 h 60-120 h 120+ h	3 (42.9%) 0 4 (57.1%)	7 (63.6%) 2 (18.2%) 2 (18.2%)	- - -	- - -
*Window Duration*				
0-2 h 2-4 h 4+ h	2 (28.6%) 2 (28.6%) 3 (42.9%)	5 (45.5%) 1 (9.1%) 5 (45.5%)	- - -	- - -
Air Quality/Odor Complaint*				
Yes No	3 (42.9%) 4 (57.1%)	6 (54.5%) 5 (45.5%)	0.13	0.35
*Recent Home Renovation*				
Yes No	3 (42.9%) 4 (57.1%)	4 (36.4%) 7 (63.6%)	0.66	0.81

Given the low completion rates for the activity logs, the following filtering steps were used: Activity logs were only included in the analysis if at least 5 of 7 days were completed. For each question, there must be a 40% or higher response rate, with at least 40% of responses in the affirmative (e.g., not “no” or “0”). For each sampler (stationary or wristband), the average PAH concentration was calculated, to assess the potential influence of various behaviors on PAH concentrations (e.g., use of heat, use of AC, etc.) * Indicates answers identified via thematic analysis. In total, 11 WB participants and 7 stationary participants completed the daily activity log. *P*-values were obtained using Welch’s t-tests for yes or no answers and utilizing the average PAH concentrations for participants.

### Health risk assessment

A risk assessment was conducted, assessing both cancer and non-cancer risk using exposure to ambient naphthalene. Naphthalene values from the stationary air samplers were used in a human health risk assessment using the same methods and values that were employed in the 2007 Public Health Consultation performed by the Oregon Department of Human Services Superfund health investigation and education program [1]. Briefly, the California Department of Environmental Quality unit risk value of 3.40E-05 was multiplied by the time weighted average air concentration at each site to yield the estimated cancer risk value [18]. The time weighted average air concentration was divided by the US EPA IRIS reference concentration of 3.00E-3 mg/m^3^ to yield the hazard quotient for each sampling site [1].

### Statistical analysis

Statistical analyses were performed using GraphPad Prism software version 10.1.2 (Boston, MA, USA) and using the R software. Concentrations for stationary samplers were converted into ng/m^3^, and all chemicals for WBs were converted into nmole/g WB. Concentrations below the instrument limit of detection (LOD) were substituted with a value equal to LOD divided by two (LOD/2). Analytes that were detected in 25% or less of samples were removed from analysis to focus on common chemical exposure results.

Descriptive statistics were calculated for individual PAHs, PAHs associated with creosote, and the average of all detected PAHs (all detected PAHs in a single sample are summed, and then the average concentration across all samples is calculated). ANOVA tests and data visualizations were performed in GraphPad Prism to assess the difference in PAH exposure by smoking status, cardinal direction, and sampling rings. Linear regression analyses were also completed in GraphPad Prism to evaluate the relationship between stationary samplers and distance from facility, WB samplers and distance from facility, as well as a direct WB and Stationary sampler comparison. For the stationary-WB comparison, stationary concentrations were log10 transformed for data visualization purposes.

To evaluate potential spatial correlation among the locations with respect to PAH concentrations, we first extracted the residuals from the linear regression models for sum PAHs and examined whether geographically proximate sites tended to have similar residuals. To do this, we plotted the pairwise distances between locations against the pairwise differences in residuals (Fig. S4). The lack of a clear increasing trend in these plots suggests no obvious spatial correlation.

In addition, we applied a Gaussian linear model with linear covariance structure to incorporate spatial correlation in a linear model that takes PAH concentrations as the response and distance from the facility as an explanatory variable [19]. This model features a spatial correlation structure in which the overall strength of the correlation is determined by the data, and the covariance in PAH concentrations between two locations decreases exponentially with the squared distance as the locations become more distant. The model was fitted using the *regress* package in R and a likelihood ratio test was conducted to evaluate the statistical significance of the spatial correlation [19]. Consistently across models, no significant spatial pattern was identified *p* > 0.99 for stationary samplers with an influential NE1 included *p* = 0.076 for stationary samplers without NE1, *p* = 0.94 for WB samplers with NE1 included, and *p* > 0.99 for WB samplers without NE1. Based on these results, we conclude that spatial correlation does not pose a concern for the validity of the decreasing trend in PAH concentrations with distance observed in the linear models.

### Report back of community-level and individual-level results

#### Development of report

An individual, personalized PAH report was previously developed with a community concerned about industrial air pollution [20], and refined in a second urban community concerned about perinatal exposure to industrial air pollution [21]. Broadly, after development, the reports were screened using the CDC Clear Communication Index [22], the Flesch Kincaid Grade Level Score (desired level of 8^th^ grade; scores ranged from 6.6–12.4) and the Flesch Reading Ease formula (desired score of >60%; scores ranged from 45–53) [23] using the built-in tools in Microsoft Word. The Clear Communication Index uses evidence-based communication strategies to score communication products, while the Flesh Kinkaid and Flesch Reading Ease metrics are commonly used measures of readability in health care [24]. The report consisted of 7 pages including a community report, individual data, full individual results with graphs, infographics on PAHs and exposures, instructions for interpreting results and suggestions for limiting PAH exposures. The community report provided a brief study overview and interpretation of the aggregated, de-identified data, comparison data, and data by distance and by direction (SI Appendix A). Individual reports showed individual data points within the context of the study population, as well as information on potential sources of PAHs, and a table listing all chemical detections within the individual’s sampler (SI Appendix B-C). Infographics regarding PAHs impact on human health, potential sources of PAHs, and mitigation strategies, were also included. Three versions of the individual report were developed: all participants received a community report, while the cover letter and individual results report differed based on which sampler type(s) the participants used in the study (WB only, stationary only, WB and stationary). The report was revised two times prior to dissemination with Beyond Toxics staff. Revisions were minimal and mostly pertained to formatting, word choice, and use of infographics to decrease large blocks of text.

#### Report dissemination

Reports were developed in SQL Server Reporting Services (SSRS) and generated programmatically using a customized python script queuing individualized report instances from a csv list of participants, utilizing the SSRS web server to save each report as a PDF. SSRS and its associated web server are directly connected to a Laboratory Information Management System (LIMS) that houses all participant ID’s and associated metadata and environmental exposure data. PDF reports were developed and personalized with name and report format selected based on participant and sampler used (e.g., WB only, stationary sampler only, WB and stationary sampler). Reports were then emailed or mailed to participants according to participant preference, and de-identified, aggregated results were also presented at multiple community meetings in collaboration with Beyond Toxics.

## Results

### Population characteristics

Participant characteristics are summarized in Table 1. Participants were 100% White, with a majority identifying as female (15/17) and between the ages of 36–65 (11/17). Limited income information was provided, although the average income of the study population is aligned with the reported per capita income of West Eugene, which is $30,293 according to the US Census Bureau from 20 02. Most participants had less than a bachelor’s degree (11/17). A minority of participants reported being cigarette smokers (6/17).

### PAH emissions

There were 38 PAHs detected in over 25% of the stationary samplers and 23 of these were detected in every sampler (Table S4). Of the creosote relevant PAHs, all 26 were detected in stationary samplers (Table S2, S4).

There were 18 PAHs detected in at least 25% of the wristband samplers, and of those, eight were found in 100% of WBs (Table S5). Over half of the PAHs detected in the wristband (*n* = 13) were considered creosote relevant (Table S2).

### Concentration as a function of distance

Our central hypothesis was based on community experience, which had anecdotally observed decreasing odors as a function of increasing distance from the facility. Therefore, we looked at PAH levels as a function of distance, across total PAH load, and individual PAHs, in both the stationary samplers, and the wristbands.

**Fig. 1:**
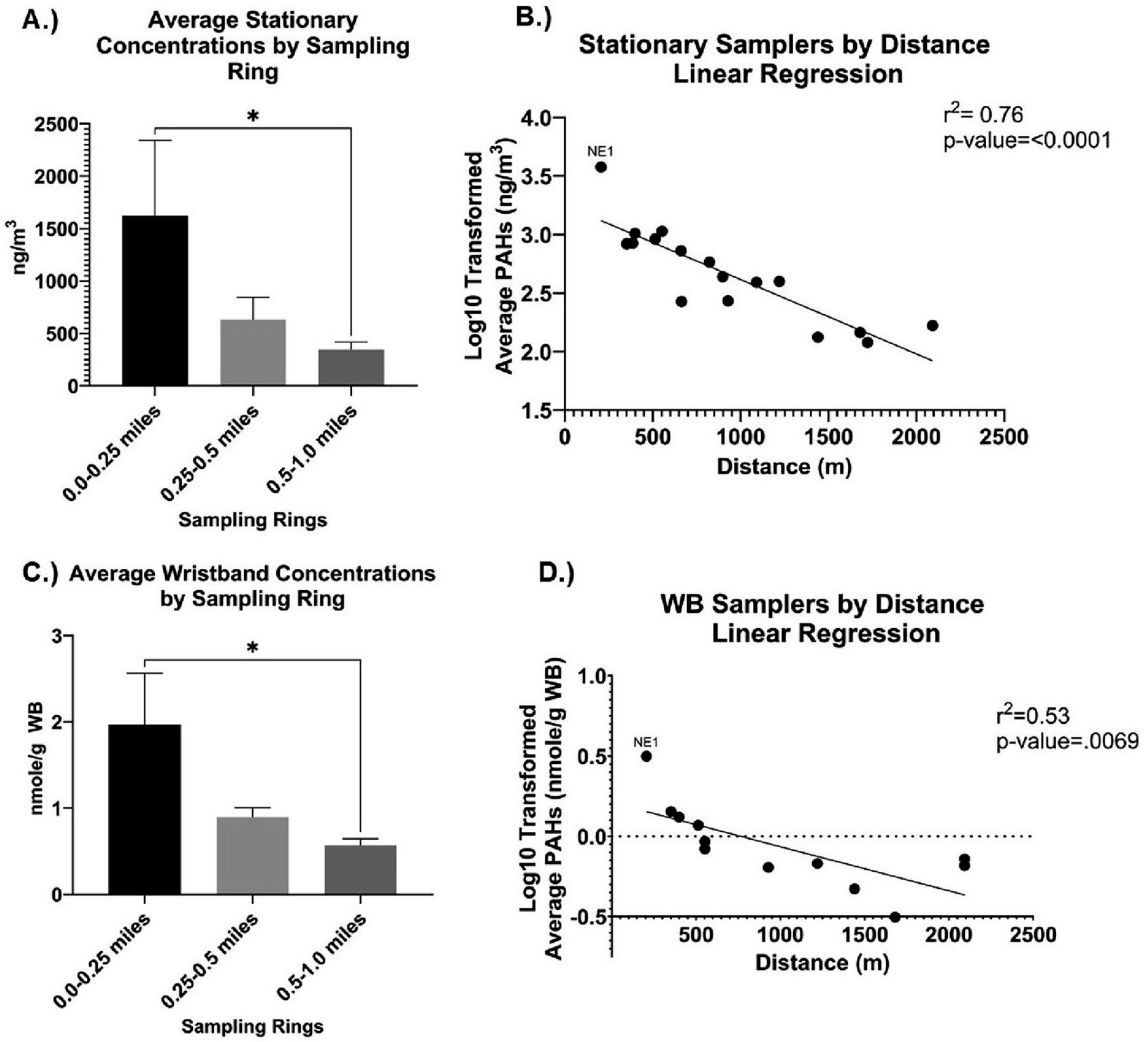
PAHs included in analysis were detected in 25% or more of samplers (one-way ANOVA *p*-value < 0.05). When a PAH was not detected, the LOD/2 was imputed.**; A** PAH concentrations stratified by sampling ring (one-way ANOVA, *p* < 0.05). **B** Values were log transformed to further assess the correlation between distance and PAH concentration (r^2^ = 0.76, *p*-value < 0.0001). **C** Average wristband concentrations stratified by sampling ring. **D** Values were log transformed to further assess the relationship between distance and PAH concentrations (r^2^ = 0.53, *p*-value = 0.0069).

When assessing distance based by sampling rings (0.25, 0.5, 1.0 miles), stationary samplers exhibited a significant decrease in PAH concentration as distance from the wood preservation facility increased (*p* < 0.05, one-way ANOVA) (Fig. 1A). For a more robust assessment, the precise location of each sampler was plotted relative to concentration, revealing a strong negative correlation between average PAH concentrations and distance, with PAH concentrations decreasing as distance from the facility increased (r^2^ = 0.76, *p*-value < 0.0001; Fig. 1B). However, one location (NE1) appeared to have a very strong influence (Fig. 1B, see notation). The location and survey responses were reviewed to determine if there were any known point sources (e.g., backyard burning, proximity to a local industrial source) to potentially explain the high PAH concentrations, yet there were no obvious explanations. As such, the values appear to be reflective of ambient exposure. However, to assess the influence of this (site)in a small data set, all analyses were run with, and without, this potentially influential data point (Table S6 and S7). With the removal of NE1, PAH concentrations were still significantly higher closest to the facility (Fig. S5A), and the correlation between distance and average PAH concentration was maintained (*p*-value = <0.0001, r^2^ = 0.76; Figure S5B).

Looking specifically at creosote relevant PAHs, the majority (15/27) trended towards highest concentrations in the inner sampling ring with 13/27 having statistically higher concentrations in the inner-most sampling ring (Fig. S6A; Table S6). When NE1 is removed the trend remains, although significance is lost following a Bonferroni-Hochberg procedure (Table S6).

Residents living within 0.25 miles of the facility had elevated levels of PAHs within their wristbands relative to those living further away (Fig. 1C). There was a strong negative correlation between wristband average PAH concentration and increasing distance from the facility (r^2^ = 0.53, *p* = 0.0069; Fig. 1D), and the correlation slightly weakened when the influential point NE1 was removed (r^2^ = 0.51, *p*-value = 0.0142; Fig. S7A). PAH concentrations were significantly higher in the inner ring and decreased to the lowest concentrations in the outer ring (Fig. 1C). This trend can be seen across the 17 detected PAHs for each wristband (Fig. S9). Of the 17 PAHs, 41.2% (n = 7) had significantly higher PAH concentrations in the inner ring (Fig. S9). When data from NE1 was removed, the trend remained, with PAH concentrations statistically highest in the inner ring and a moderate trend for PAH concentrations decreasing as distance from the facility increased (Fig. S7B).

Of the PAHs detected in the wristband, 14 were identified as creosote relevant PAHs (Table S2) and were detected across all wristband samplers. Ten of the creosote-relevant PAHs showed a trend for elevated PAH concentrations in the inner ring (Table S7; Fig. S9); seven were statistically significant (Table S7; Fig. S9). When the influential point was removed, five of the seven PAHs remained significantly different: 2-methylnaphthalene, naphthalene, 1-methylnaphthalene, fluorene, and 2-methylnaphthalene (Table S7).

### Comparison between stationary and wristband samplers

**Fig. 2:**
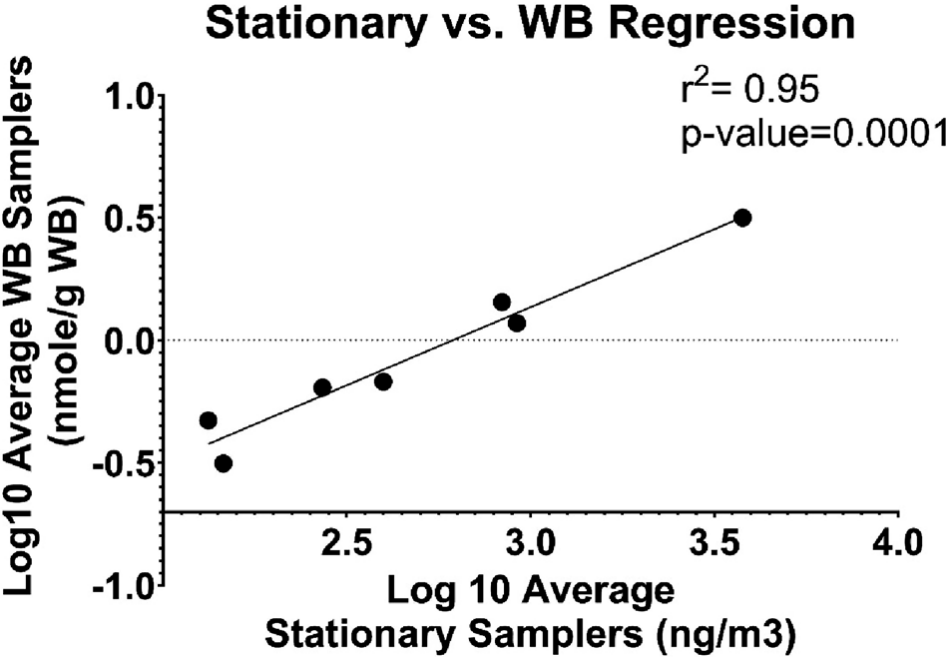
Regression comparing stationary and WB samplers. Values were log transformed to further assess the correlation between sampler type (r^2^ = 0.95, *p*-value = 0.0001).

Within the dataset, there were eight paired samplers, wherein a stationary sampler was set up at a location where one or more individuals also wore a silicone wristband. This enabled an assessment of PAH concentrations between environmental and personal exposures. Sum PAH concentrations were log10 transformed for both sampler types to normalize the data and paired values were plotted against each other to assess the potential correlation. There is a strong correlation (r^2^ = 0.95 *p*-value = 0.0001) between the samplers; as PAH concentrations increase in the stationary samplers, they also increase in the wristbands (Fig. 2). The correlation between stationary and WB samplers remained even when NE1 data was removed from both the stationary and WB datasets (*p*-value = 0.0040, r^2^ = 0.90; Fig. S10).

While the stationary samplers had a greater number of detected PAHs (*n* = 37) relative to the WB (*n* = 17), the five most predominant PAHs by average concentration were naphthalene, acenaphthene, 1-methylnaphthalene, 2-methylnaphthalene and fluorene for stationary samplers (Fig. S11A). This was similar for the WB, with the only difference being that in place of 1-methylnaphthalene, the WB had phenanthrene in the top five most predominant PAHs (Fig. S11B).

### PAH exposure as a function of cardinal direction

**Fig. 3:**
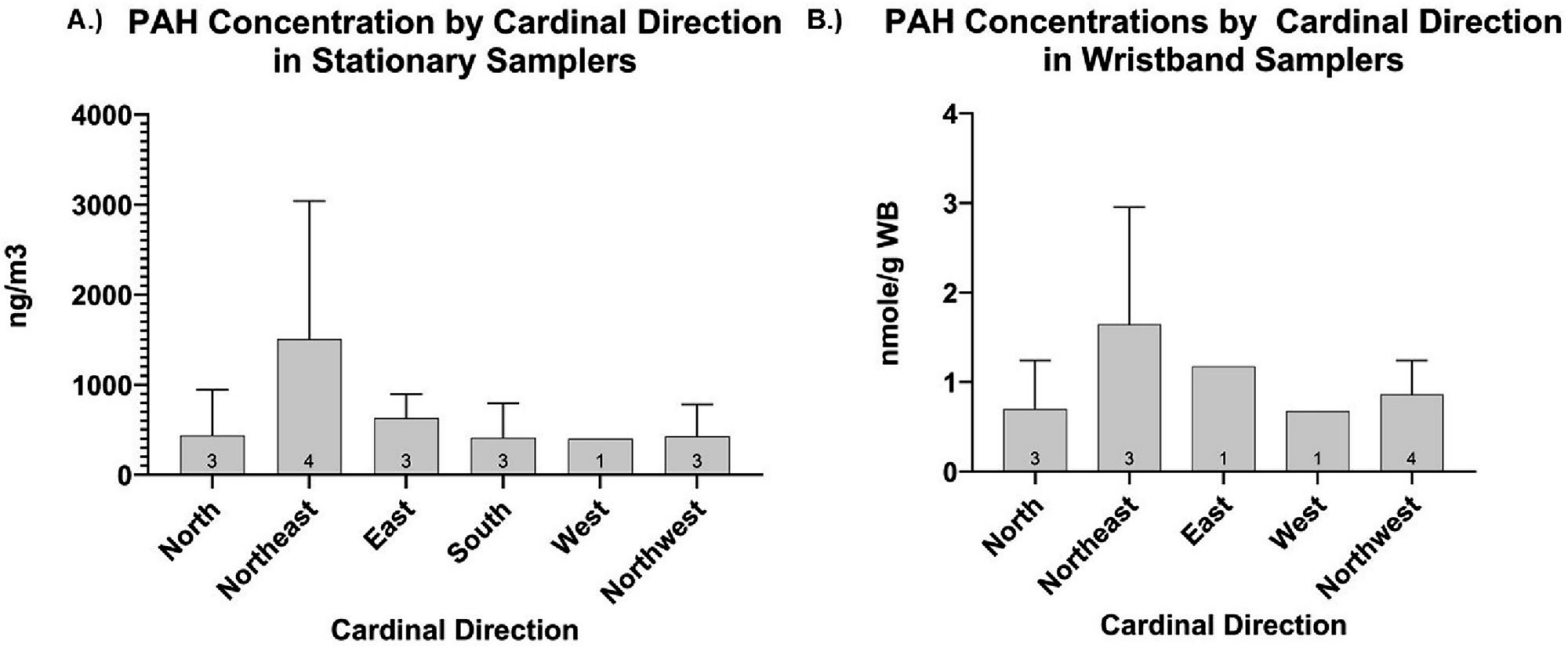
PAH concentration in samplers stratified by cardinal direction. **A** Stationary samplers. **B** WB samplers. The average PAH concentration, by cardinal distance, was calculated. The number of samplers is displayed within each bar. One-way ANOVA, *p*-value < 0.05.

Anecdotal evidence from community observations indicates that odors, while most detectable in the northeast, downwind of the facility, were also detected in the east. This is supported by decades of odor complaints [25]. Therefore, we investigated the relationship between cardinal direction and PAH levels detected in the stationary and WB samplers. Across all stationary samplers, concentrations were highest in the northeast, followed by the east. (Fig. 3A). Locations for WBs were obtained through addresses provided by participants; however, this is an imperfect substitute as the wristbands were worn, and people were mobile (e.g., leaving home to go to work, run errands, recreate, etc.) throughout the duration of the study. Concentrations were also highest in the northeast, and the east (Fig. 3B).

### Co-variates

All participants completed an initial questionnaire for enrollment (Table S3) and a daily activity log (Table 2). A total of 11 WB participants and 7 stationary participants completed the activity log, while all participants included in the final data analysis completed the questionnaire. Questionnaire and activity log data were filtered to include only items with a minimum response rate of 40%, as well as a minimum affirmative response rate of 40% (i.e., ≥40% of respondents selected an affirmative answer such as “yes” or a non-zero value). Based on these criteria, the covariates included in the analysis were recent home renovations, duration of electric heat use, duration of AC or air filtration use, duration of open window use, and odor complaints. Sum PAH concentrations were calculated for individual samplers and compared across covariates to assess whether reported behaviors were associated with elevated concentrations; however, no statistically significant associations were observed (Table 2). Due to the limited sample size, statistical analyses were restricted to questionnaire items with binary response options; Welch’s t-tests were conducted for these items, while questions with multiple categorical responses were excluded from statistical testing (Table 2). The questions included with binary responses were pertaining to reported odor and recent home renovations. Both questions did not result in any statistical significance for either sampler when comparing sum PAH concentrations amongst participants who answered yes and no.

One of the covariates with the highest response rate (15/17 participants), was reporting smelling odors that resemble moth balls, a strong chemical smell, or industrial air toxics. Despite decades of community odor complaints, which were often attributed to naphthalene, the naphthalene concentrations detected were well below the odor threshold. The lowest published concentration of naphthalene found to elicit odor was 7500 ng/m^3^[26], while the odor threshold is at 0.44 mg/m^3^ (440,000 ng/m^3^) However, creosote is a mixture, and thus the odors that the community reported, frequently described as ‘toxic air’, ‘creosote smell’ and ‘heavy car exhaust’, are likely reflective of the mixture, and not a single chemical. While the two participants who did not express smelling these odors regularly did have lower sum PAH concentrations, there was no statistically significant finding when assessing covariate data, which could be due to small sample size (Table 2). Of interest, community members reported to Beyond Toxics that the odors were substantially reduced following the facility shutdown in February 2022.

### Health endpoints

Given the previous health consultation on naphthalene exposures, stationary naphthalene concentrations were assessed for health endpoints. The EPA inhalation reference level for naphthalene is 3000 ng/m^3^[27]. The highest individual naphthalene concentration in a stationary sampler was 846 ng/m^3^ and the average naphthalene concentration was 161 ng/m^3^; levels below the EPA inhalation reference level [27]. Naphthalene concentrations were also used to calculate cancer and non-cancer health risk. Calculating discrete site cancer and non-cancer risk values resulted in no site exceeding additional cancer cases above background cancer rates. This finding is in line with the 2007 Oregon Department of Human Services SHINE investigation findings [1]. Similarly, the calculated non-cancer endpoint values (Table S8) did not exceed levels of concern and were significantly lower than the 24-h maximum calculated hazard quotient values determined in the 2007 SHINE investigation [1].

### Comparisons to other residential and urban areas

**Fig. 4:**
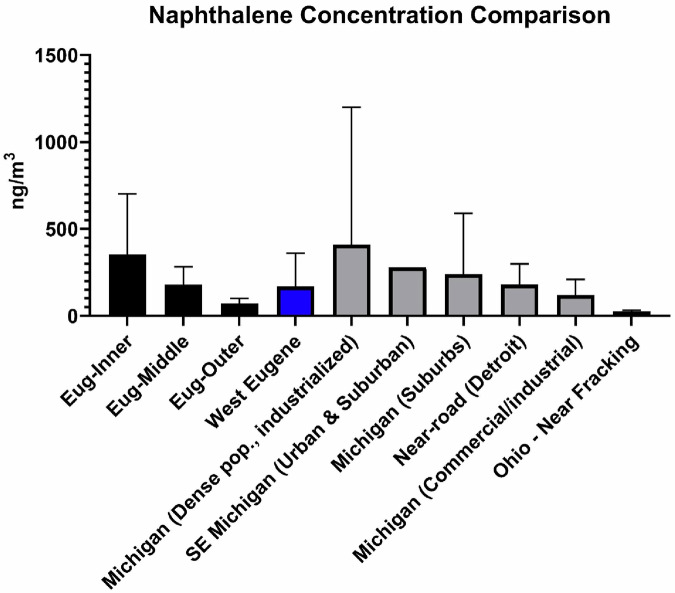
Comparison of average naphthalene concentrations in stationary samplers to other urban areas in the US. PAH concentrations by sampling ring (current study) are shown in the black bars, while the blue bar shows the average PAH concentration across all samplers. Grey bars denote average PAH concentrations from studies in Michigan, including Detroit (Batterman et al, 2012) and rural Ohio (Paulik et al, 2016; Paulik et al., 2018).

Naphthalene is a common PAH constituent in creosote [2, 17, 28, 29]. Naphthalene levels were consistent relative to other residential and urban areas across the United States (Fig. 4). Specifically, naphthalene concentrations within the stationary samplers were compared to other passive sampling data from locations in rural Ohio and Michigan (Detroit, Southeast Michigan, industrial and commercial areas, densely populated areas, and suburban areas). Levels in West Eugene were similar to that of Detroit and southeast Michigan (Fig. 4) [30, 31].

PAH concentrations in the WB are more difficult to compare, as the concentrations cannot be back calculated to air concentrations. However, detection frequency was compared to see if the PAHs that were detected from WB participants were representative of other studies in the literature. A recent review paper identified the most commonly abundant PAHs [32]. The review showed 100% detection frequency in at least one other study for all our 100% detected PAHs (Table S9). Despite not being the most abundant in the literature, 2-methylnaphthalene was the most frequently reported alkylated PAH that was detected among the studies that were reviewed. Overall, detection frequency for our study with WB samplers appears reflective of what has been found in other studies.

## Discussion

Traditionally, wood manufacturing facilities have utilized chemicals like creosote and pentachlorophenol in order to prevent damage due to insects and to slow weathering of the materials [33]. While this practice is effective in preserving wood materials, such chemicals are known for their carcinogenic potential and threat to public health [34]. Creosote, one of the most common mixtures to treat wood in manufacturing facilities, is typically derived from coal tar, and can contain up to 85% PAHs [33].

The presence of the wood manufacturing facility in West Eugene has been a concern to the surrounding communities for decades [25]. This study was conducted in response to their ongoing odor complaints and health concerns regarding proximity to known industrial air pollution sources. Broadly, the results of this study were highly aligned with community reports and anecdotal observations.

PAH concentrations were highest closest to the facility, and the highest concentrations were also found to the northeast and east, matching decades of odor complaints. Our findings also aligned with previous research on industrial and wood-preservative facilities that report elevated PAH concentrations in closer proximity to emission sources. For example, Amodio et al. (2013) found that PAH concentrations in ambient air were highest at monitoring sites downwind and adjacent to a steel manufacturing complex, indicating influence from the facility on nearby air quality [35]. Similarly, Liu et al. (2019) measured PAHs around a coke plant and observed a spatial gradient in concentrations, with levels highest on the coke side of the facility and decreasing at greater distances [36]. These observations align with the gradients detected in the current study, highlighting similar proximity-driven differences in ambient PAH levels. Notably, naphthalenes, fluorene, phenanthrene, acenaphthene and anthracene are the most commonly found PAHs when assessing pollution from wood preservative facilities - likely due to their high volatility [37]. This supports our findings, as fluorene, phenanthrene, acenaphthene and naphthalenes were the most abundant PAHs detected. Our findings also align with other studies, such as Bouchard et al. 2001- who found that residents that lived within 0.25 miles of a creosote site had elevated naphthalene levels [3]. However, creosote signatures at further distances like 0.5- and 1-mile marks have not been frequently explored in the literature.

Other studies that specifically looked at differences between indoor and outdoor air, using stationary samplers, also found household-level differences, indicating both outdoor exposures and indoor exposures [38]. For example, Ghetu et al. (2022) used paired indoor and outdoor passive samplers and observed that concentrations of PAHs varied considerably between homes. Indoor concentrations were correlated with outdoor levels, suggesting infiltration of ambient air, but differences in indoor-to-outdoor ratios indicated that building characteristics, ventilation patterns and possible indoor sources also contributed to overall household exposure [38]. In this study, however, every single PAH detected in the WB was also detected in the stationary samplers. Furthermore, the composition of the detected PAHs between sampler types was similar. This may indicate a common exposure source, that may perhaps be muffling the contribution of individual residential exposures (e.g., burning candles, use of air fresheners). In our study we also saw a strong relationship between stationary samplers co-located at a residence wherein a person is additionally wearing a WB, potentially indicating a similar exposure source or sources. This is supported by the lack of unique PAH detections within the wristband; all wristband detections were also found in the stationary sampler.

Although both samplers show a general trend of higher concentrations closest to the facility, there were differences in some creosote relevant chemicals. Because the WBs are personal samplers worn throughout the day, it is expected for the chemicals and detection concentrations to differ from the stationary samplers. Because the stationary samplers remain in the same location throughout the sampling period, they are more representative of the PAH concentrations of this area. Many of the participants with WBs reported daily commuting and driving times in their daily activity logs- indicating they were not always in the area near the facility. This could explain less creosote relevant detections and lower PAH concentrations all together. Ultimately, there seems to be an external common source of pollution indicated by the correlation between the stationary and WB samplers (Fig. 2). Detection frequency is often different between sampler type, as there are commonly higher exposures found in personal samplers as a result of poorer air quality in indoor spaces [38, 39]. We can suggest for a future point of interest that those external sources could potentially have been strong enough to override individual variation that is commonly present in WB samplers in the literature.

While community concerns were focused on the proximity to industrial emissions, we additionally assessed the potential for lifestyle factors to influence exposure. Six participants reported smoking cigarettes; however, response rates were low (Fig. S12). Of the six participants who identified as smokers, one was excluded due to incomplete survey data. Among the remaining participants, three had only stationary samplers, while two had both stationary and wristband samplers. Among these five participants, one was located northeast of the facility, two were located east, and the remaining two were located to the south. Similarly, none of the co-variates examined—smoking, ventilation practices, detection of odors, heating/cooling sources, or home renovation—were associated with PAH concentrations. We have previously noted that self-reported exposure to cigarette smoke was not correlated with the average PAH concentration in a wristband [40]. While we are unable to conclusively identify the source of the PAHs detected – PAHs are ubiquitous in the environment and are produced both naturally and through industrial processes, there was a high overlap of PAHs found in the outdoor stationary samplers and those worn by participants. This is somewhat unusual as other studies with paired stationary and wristband samples identified unique exposures in the wristband, often indicating household-level exposures [41].

We focused specifically on naphthalene, due to the high percentage found in creosote, as well as community concerns related to odor, and prior investigations that evaluated naphthalene concentrations in West Eugene. While a prior investigation found that naphthalene levels exceeded levels of health concern, these values were only found in extreme proximity to the facility, and during a time that creosote emissions were assumed to be maximal; levels in residential areas were below levels of concern [1]. Like the prior sampling events, naphthalene levels were highest closest to the facility, but no values exceeded cancer or non-cancer endpoints. Notably though, naphthalene levels were similar to densely populated urban areas, specifically showing similar concentrations as have been detected in Detroit, Michigan [30].

Our study was strengthened by the integration of community concerns, priorities and community-engaged approach at the outset, which informed the study design, recruitment, data collection, data analysis, and interpretation of results. We were able to look at correlations between stationary and wristband samplers and assess exposure to PAHs as a function of distance and direction and were able to analyze for 64 total PAHs- many of which have been associated with creosote use. Additionally, high compliance with both sampler types (92.3% WB, 94.1% stationary) strengthened data analysis and allowed for comparisons between sampling rings. As such, this study was sufficient to address community concerns, and results were returned to study individuals, and through Beyond Toxics newsletters. However, there were several limitations, predominantly the small sample size. With a larger sample size, it is possible we may have been able to determine a higher correlation between sampler types, identify a clearer relationship between sampling rings and PAH levels, and cardinal direction as well. Additionally, there were not sufficient participation rates in daily activity logs for any conclusions to be drawn pertaining to co-variates and confounding factors. For cardinal direction specifically, exposures were evaluated exclusively in response to community comments and concerns about wind patterns impacting exposures for report-back purposes. Due to the limited sample size, we were unable to make any statistically significant conclusions regarding cardinal direction or pollution sources.

Lastly, the findings of this study should be interpreted within the context of the community’s socioeconomic and environmental justice profile. The average income of participants closely mirrored the per capita income of West Eugene, reported as $30,293 by the U.S. Census Bureau in 2022—substantially lower than the national median household income by $44,287 [42]. This economic disparity highlights structural inequities that may contribute to disproportionate environmental exposures. Additionally, most participants had not attained a Bachelor’s degree, a factor commonly associated with limited access to environmental protections and public health resources (Table 1) [43, 44]. Together, these indicators underscore the importance of considering socioeconomic, demographic and educational contexts when assessing environmental health risks and crafting equitable policy interventions.

## Supplementary information

Supplementary Information

## Data Availability

The datasets generated during the current study are not publicly available due to privacy concerns but are available from the corresponding author on reasonable request. The datasets generated during and/or analyzed during the current study are available from the corresponding author on reasonable request.
