# Supplementary material for: A community-engaged investigation of residential polycyclic aromatic hydrocarbon exposures in West Eugene, OR
Source: J Expo Sci Environ Epidemiol. 2026 Apr 8;36(4):694–704. doi: 10.1038/s41370-026-00863-w (PMC13331731; doi:10.1038/s41370-026-00863-w)
Supplement: Supplementary file 1 — Supplementary Information [file 41370_2026_863_MOESM1_ESM.docx]

Contents

[TABLES 3](#_Toc211684981)

[Table S1. List of QC and target PAHs in GC-MS/MS method 3](#_Toc211684982)

[Table S2. Detection of creosote relevant PAHs according to sampler type 6](#_Toc211684983)

[Table S3. Survey questions and possible answers posed to the participants with accompanied data dictionary used for analysis. 7](#_Toc211684984)

[Table S4. PAHs detected in stationary samplers. 11](#_Toc211684985)

[Table S5. PAHs detected in WB samplers. 13](#_Toc211684986)

[Table S6. Creosote relevant stationary PAHs comparison with and without NE1 influential point 14](#_Toc211684987)

[Table S7. Creosote relevant WB PAHs comparison with and without NE1 influential point 15](#_Toc211684988)

[Table S8. Hazard quotient and Cancer risk assessment for naphthalene. 16](#_Toc211684989)

[Table S9. Comparison of detection frequency between chemicals found in WBs and other studies using WB samplers 16](#_Toc211684990)

[FIGURES 18](#_Toc211684991)

[Figure S1. Instructional information regarding WB and stationary setup and use instructions 18](#_Toc211684992)

[Figure S2. Final study design and layout of samplers in relation to the wood preservation facility. 18](#_Toc211684993)

[Figure S3. Stationary sampler setup on residential participant property. 19](#_Toc211684994)

[Figure S4. Pairwise distances between locations against differences in residuals for spatial analysis 20](#_Toc211684995)

[Figure S5. PAH detection in stationary samplers according to distance without the presence of influential sampler NE1 20](#_Toc211684996)

[Figure S6. Creosote-relevant PAHs in samplers. 22](#_Toc211684997)

[Figure S7. PAH detection in WB samplers according to distance without the presence of influential sampler NE1 23](#_Toc211684998)

[Figure S8. PAHs by sampling ring in stationary samplers. 24](#_Toc211684999)

[Figure S9. PAHs by sampling ring in WB samplers. 24](#_Toc211685000)

[Figure S10. Correlation between stationary and WB samplers with influential sampler NE1 removed 26](#_Toc211685001)

[Figure S11. Percent of total sum PAH concentrations for stationary (A) and WB (B) samplers 27](#_Toc211685002)

[Figure S12. Average PAH concentrations according to sampling rings (0.25, 0.5, 1.0 miles) for participants identifying as smokers 27](#_Toc211685003)

[APPENDICES 28](#_Toc211685004)

[Appendix A. Community-level report - 4 pages 29](#_Toc211685005)

[Appendix B. Anonymized individual stationary sampler report – 7 pages. 33](#_Toc211685006)

[Appendix C. Anonymized individual wristband sampler report – 7 pages. 40](#_Toc211685007)

[CALCULATIONS 47](#_Toc211685008)

# TABLES

Table S1. List of QC and target PAHs in GC-MS/MS method. Performance reference compounds (PRCs), internal standard (IS), surrogates, and target polycyclic aromatic hydrocarbons (PAH) are given for the GC-MS Triple Quad method used for PAH analysis in this study, with instrument limits of detection (LOD) and limits of quantitation (LOQ).

| **PAH** | **Category** | **CAS #** | **LOD (ng/mL)** | **LOQ (ng/mL)** |
| --- | --- | --- | --- | --- |
| Fluorene-d10 | PRC | 81103-79-9 | 0.33 | 1.0 |
| Pyrene-d10 | PRC | 1718-52-1 | 0.42 | 2.1 |
| Benzo[b]fluoranthene-d12 | PRC | 205-99-2 | 1.7 | 5.0 |
| Perylene-d12 | IS | 1520-96-3 | 1.7 | N/A |
| Naphthalene-d8 | Surrogate | 1146-65-2 | 0.33 | 1.0 |
| Acenaphthylene-d8 | Surrogate | 93951-97-4 | 0.33 | 1.0 |
| Phenanthrene-d10 | Surrogate | 1517-22-2 | 1.7 | 5.0 |
| Fluoranthene-d10 | Surrogate | 93951-69-0 | 1.7 | 5.0 |
| Chrysene-d12 | Surrogate | 1719-03-5 | 1.7 | 5.0 |
| Benzo[a]pyrene-d12 | Surrogate | 63466-71-7 | 1.7 | 5.0 |
| Benzo[ghi]perylene-d12 | Surrogate | 93951-66-7 | 1.7 | 5.0 |
| Naphthalene | Target | 91-20-3 | 1.0 | 5.2 |
| 2-Methylnaphthalene | Target | 91-57-6 | 0.70 | 3.5 |
| 1-Methylnaphthalene | Target | 90-12-0 | 0.28 | 1.4 |
| 2-Ethylnaphthalene | Target | 939-27-5 | 0.97 | 4.8 |
| 2,6-Dimethylnaphthalene | Target | 581-42-0 | 0.89 | 4.4 |
| 1,6-Dimethylnaphthalene | Target | 575-43-9 | 0.81 | 4.1 |
| 1,4-Dimethylnaphthalene | Target | 571-58-4 | 1.2 | 6.2 |
| 1,5-Dimethylnaphthalene | Target | 571-61-9 | 1.2 | 5.9 |
| 1,2-Dimethylnaphthalene | Target | 573-98-8 | 0.94 | 4.7 |
| 1,8-Dimethylnaphthalene | Target | 569-41-5 | 0.83 | 4.2 |
| 2,6-Diethylnaphthalene | Target | 59919-41-4 | 0.81 | 4.1 |
| Acenaphthylene | Target | 208-96-8 | 2.3 | 12 |
| Acenaphthene | Target | 83-32-9 | 1.1 | 5.4 |
| Fluorene | Target | 86-73-7 | 0.79 | 4.0 |
| Dibenzothiophene | Target | 132-65-0 | 0.24 | 1.2 |
| Phenanthrene | Target | 85-01-8 | 0.46 | 2.3 |
| Anthracene | Target | 120-12-7 | 1.1 | 5.2 |
| 2-Methylphenanthrene | Target | 2531-84-2 | 0.39 | 1.9 |
| 2-Methylanthracene | Target | 613-12-7 | 0.47 | 2.4 |
| 1-Methylphenanthrene | Target | 832-69-9 | 1.1 | 5.3 |
| 9-Methylanthracene | Target | 779-02-2 | 0.87 | 4.4 |
| 3,6-Dimethylphenanthrene | Target | 1576-67-6 | 0.42 | 2.1 |
| 2,3-Dimethylanthracene | Target | 613-06-9 | 0.34 | 1.7 |
| Fluoranthene | Target | 206-44-0 | 0.54 | 2.7 |
| 9,10-Dimethylanthracene | Target | 781-43-1 | 0.85 | 4.2 |
| Pyrene | Target | 129-00-0 | 0.42 | 2.1 |
| Retene | Target | 483-65-8 | 0.84 | 4.2 |
| Benzo[a]fluorene | Target | 238-84-6 | 1.7 | 5.0 |
| Benzo[b]fluorene | Target | 243-17-4 | 1.7 | 5.0 |
| Benzo[c]fluorene | Target | 205-12-9 | 0.30 | 1.5 |
| 1-Methylpyrene | Target | 2381-21-7 | 0.38 | 1.9 |
| Benz[a]anthracene | Target | 56-55-3 | 0.75 | 3.8 |
| Cyclopenta[c,d]pyrene | Target | 27208-37-3 | 0.53 | 2.7 |
| Triphenylene | Target | 217-59-4 | 0.41 | 2.0 |
| Chrysene | Target | 218-01-9 | 0.50 | 2.5 |
| 6-Methylchrysene | Target | 1705-85-7 | 0.89 | 4.4 |
| 5-Methylchrysene | Target | 3697-24-3 | 1.7 | 5.0 |
| Benzo[b]fluoranthene | Target | 205-99-2 | 0.37 | 1.9 |
| 7,12-Dimethylbenz[a]anthracene | Target | 57-97-6 | 0.94 | 4.7 |
| Benzo[k]fluoranthene | Target | 207-08-9 | 0.53 | 2.6 |
| Benzo[j]fluoranthene | Target | 205-82-3 | 0.56 | 2.8 |
| Benz[j]&[e]aceanthrylene | Target | 202-33-5 and 199-54-2 | 1.7 | 5.0 |
| Benzo[e]pyrene | Target | 192-97-2 | 0.71 | 3.5 |
| Benzo[a]pyrene | Target | 50-32-8 | 1.2 | 5.9 |
| Indeno[1,2,3-c,d]pyrene | Target | 193-39-5 | 0.26 | 1.3 |
| Dibenzo[a,h]pyrene | Target | 53-70-3 | 1.0 | 5.1 |
| Picene | Target | 213-46-7 | 0.74 | 3.7 |
| Benzo[ghi]perylene | Target | 191-24-2 | 0.34 | 1.7 |
| Anthanthrene | Target | 191-26-4 | 0.33 | 1.7 |
| Naphtho[1,2-b]fluoranthene | Target | 5385-22-8 | 1.7 | 5.0 |
| Naphtho[2,3-j]fluoranthene | Target | 205-83-4 | 1.7 | 5.0 |
| Dibenzo[a,e]fluoranthene | Target | 5385-75-1 | 0.47 | 2.4 |
| Dibenzo[a,l]pyrene | Target | 191-30-0 | 0.48 | 2.4 |
| Naphtho[2,3-k]fluoranthene | Target | 207-18-1 | 1.7 | 5.0 |
| Naphtho[2,3-e]pyrene | Target | 193-09-9 | 1.7 | 5.0 |
| Dibenzo[a,e]pyrene | Target | 192-65-4 | 6.4 | 32 |
| Coronene | Target | 191-07-1 | 0.7 | 3.5 |
| Dibenzo[e,l]pyrene | Target | 192-51-8 | 1.7 | 5.0 |
| Naphtho[2,3-a]pyrene | Target | 196-42-9 | 1.7 | 5.0 |
| Benzo[b]perylene | Target | 197-70-6 | 1.7 | 5.0 |
| Dibenzo[a,i]pyrene | Target | 189-55-9 | 1.4 | 7.1 |
| Dibenz[a,h]anthracene | Target | 189-64-0 | 0.52 | 2.6 |

Table S2. Detection of creosote relevant PAHs according to sampler type. Sources identifying PAH creosote relevance are noted.

| **Creosote Relevant PAHs** | **Stationary** | **WB** | **Source** |
| --- | --- | --- | --- |
| 1,2-Dimethylnaphthalene | ***** | ***** | 1, 2. |
| 1,4- Dimethylnaphthalene | ***** |  | 1, 2. |
| 1,5- Dimethylnaphthalene | ***** |  | 1, 2. |
| 1,6 and 1,3- Dimethylnaphthalene | ***** | ***** | 1, 2. |
| 1,8- Dimethylnaphthalene | ***** |  | 1, 2. |
| 1-Methylnaphthalene | ***** | ***** | 2, 3, 4, 5. |
| 2,6-Dimethylnaphthalene | ***** | ***** | 1, 2. |
| 2-Methylnaphthalene | ***** | ***** | 2, 3, 4, 5. |
| 2-Methylphenanthrene | ***** | ***** | 2, 3. |
| Acenaphthene | ***** | ***** | 2, 5, 6, 7, 8. |
| Acenaphthylene | ***** |  | 2, 4, 5, 8. |
| Anthracene | ***** |  | 2, 5, 8, 9. |
| Benz[a]anthracene | ***** |  | 2, 3, 4, 5, 8. |
| Benzo[a]fluorene | ***** |  | 2, 3. |
| Benzo[a]pyrene | ***** |  | 2, 4, 8. |
| Benzo[b]fluoranthene | ***** |  | 2, 3, 8. |
| Benzo[b]fluorene | ***** |  | 2, 3. |
| Benzo[e]pyrene | ***** |  | 2, 3, 4, 5. |
| Benzo[g,h,i]perylene | ***** |  | 2, 3. |
| Benzo[k]fluoranthene | ***** |  | 2, 4, 5, 8. |
| Chrysene | ***** |  | 2, 8, 10. |
| Dibenzothiophene | ***** | ***** | 2, 11. |
| Fluoranthene | ***** | ***** | 2, 8, 12. |
| Fluorene | ***** | ***** | 2, 8,13, 14. |
| Naphthalene | ***** | ***** | 2, 7, 8, 14. |
| Phenanthrene | ***** | ***** | 2, 8. 15. |
| Pyrene | ***** | ***** | 2, 8, 15. |
| Retene | ***** | ***** | 2, 14. |
| ^1^(Nestler, 1974), ^2^(NTP, 2021), ^3^(Murphy & Brown, 2005), ^4^(Agency for Toxic Substances and Disease Registry, 2023), ^5^(WHO, 2004), ^6^(*Substance Information - ECHA*, n.d.), ^7^(Mueller et al., 1989), ^8^(Gallego et al., 2008), ^9^(US EPA, n.d.), ^10^(Biswas & Ghosh, 2014), ^11^(PubChem, n.d.), ^12^(Bos et al., 1987), ^13^(Alhamdow et al., 2020),^14^(Rivera et al., 2022), ^15^(Hultgren et al., 2010). | | | |

## Table S3. Survey questions and possible answers posed to the participants with accompanied data dictionary used for analysis.

| Question | Value |  | Variable |
| --- | --- | --- | --- |
| Please check what age group applies to you (the person filling out the study) | 1 2 3 4 |  | 0-17 years  18-35 years  36-65 years 66+ years |
| Would you like to participate in this study? By selecting yes you indicate that you understand the study and that you agree to take part in this study. | 1 0 |  | Yes No |
| Which of the following activities would you like to participate in as part of the West Eugene Air Quality study? | 1 2 3 999 |  | Silicone wristband  Stationary air monitor WB and stationary  None of the above |
| Who are you filling this survey out for? | 1 2 3 4  5 |  | Myself My child (age 5-17)  My child (age 5-17) * I am their legal guardian Someone else |
| Please provide your full name | --- |  | n/a |
| Would your child like to be part of this research study? | 1 0 |  | Yes No |
| Please provide your child's full name (First, Last) | --- |  | n/a |
| Earlier we described the study and what you would need to do. Please mark the study activities you and/or your child would be asked to participate in: | 1  2  3 |  | Wear a wristband and/or host a stationary air sampler on your property AND fill out questionnaires  Take photographs of air pollution AND water samples Take water samples |
| Would you like to receive results? | 1 0 |  | Yes No |
| May we use your samples in future studies? | 1 0 |  | Yes No |
| May we contact you again for related research? | 1 0 |  | Yes No |
| Please provide your home street address | --- |  | n/a |
| Please provide your telephone number and/or email | --- |  | n/a |
| What is your race? | 1 2 3 4 5 6 7 999 |  | White  Black or African American American Indian or Alaska Native Asian Native Hawaiian or Pacific Islander  More than one race Unknown or other Prefer not to answer |
| What is your ethnicity? | 1 2 999 |  | Not Hispanic or Latino Hispanic or Latino  Prefer not to answer |
| What is your gender? | --- |  | n/a |
| What is your highest level of education you have completed? | 1 2 3 4 5 6 7 999 |  | 8th grade High school Some college  Associates degree  Bachelor's degree  Master's degree Doctoral degree  Prefer not to answer |
| What is your current income level of your household? | 1 2 3 4 5 6 7 8 9 999 |  | Under $9.999 $10,000-$19,999 $20,000-$29,999 $30,000-$39,999 $40,000-$49,999 $50,000-$59,999 $60,000-$69,999 $70,000-$79,999 $80,000 or more Prefer not to answer |
| When was your house/business built? | --- |  | n/a |
| Is the sampling location a primary residence? | 1 0 |  | Yes No |
| Have you smelled odors that resemble moth balls, a strong chemical smell, or industrial air toxics when you are home? | 1 0 |  | Yes No |
| Approximately how long do you smell these odors? | --- |  | n/a |
| What type of flooring do you have in your house? Select the major type of flooring. | 1 2 3 4 5 999 |  | Hardwood Carpet Vinyl Laminate Other Prefer not to answer |
| Prior to this study, did you have installations or remodeling done in your home/business? For example, painting, laminate flooring, cabinet install, tile, new carpeting, etc. | 1 0 999 |  | Yes No Unsure |
| How recently did this remodeling occur? | 1 2 3 4 999 |  | 0-3 months 4-6 months 7-9 months 10+ months Unsure |
| Please state what kind of remodeling was done (addition, add a deck, new roof, insulation, etc.) | --- |  | n/a |
| Do you have any hobbies which include the use of glues and adhesives, wood preservatives, or paints? | 1 0 |  | Yes  No |
| Do you conduct these hobbies inside your home or business? | 1 0 999 |  | Yes  No Prefer not to answer |
| How many times a month do you vacuum? | 1 2 3 4 5 999 |  | 0-5 times a month 6-11 times a month 11-15 times a month 15 or more times a month Not applicable  Prefer not to answer |
| How many times a month do you mop? | 1 2 3 4 5 999 |  | 0-5 times a month 6-11 times a month 11-15 times a month 15 or more times a month Not applicable  Prefer not to answer |
| Do you use air fresheners? | 1 0 |  | Yes No |
| Approximately how many air fresheners do you use? | 1 2 3 4 999 |  | 0-5  6-11 11-15 15 or more Prefer not to answer |
| Have chemicals been applied in your home/business to kill insects, either recently or since you have occupied the home/business? | 1 0 2 999 |  | Yes  No I don't know Prefer not to answer |
| Have pesticides ever been applied outside of your home/business? | 1 0 2 999 |  | Yes  No I don't know Prefer not to answer |
| Do you have a gas stove? | 1 0 2 999 |  | Yes  No Prefer not to answer |
| Do you or anyone in your home/business smoke? | 1 0 2 999 |  | Yes  No Prefer not to answer |
| Do they smoke inside or outside your home/business? | 0 1 2 999 |  | Inside Outside I don't know Prefer not to answer |

## Table S4. PAHs detected in stationary samplers.

| **Chemical Name** | **Average Concentration (ng/m^3^)** | **Detection Frequency** |
| --- | --- | --- |
| 1,2-Dimethylnaphthalene | 4.72 | 100% |
| 1,4-Dimethylnaphthalene | 2.58 | 100% |
| 1,5-Dimethylnaphthalene | 3.09 | 100% |
| 1,6 and 1,3-Dimethylnaphthalene | 15.34 | 100% |
| 1-Methylnaphthalene | 87.01 | 100% |
| 2,6- Dimethylnaphthalene | 23.67 | 100% |
| 2-Ethylnaphthalene | 9.75 | 100% |
| 2-Methylanthracene | 0.84 | 100% |
| 2-Methylnaphthalene | 159.58 | 100% |
| 2-Methylphenanthrene | 1.68 | 100% |
| Acenaphthene | 165.41 | 100% |
| Acenaphthylene | 1.82 | 100% |
| Anthracene | 1.90 | 100% |
| Benz[a]anthracene | 0.05 | 100% |
| Benzo[ghi]perylene | 0.01 | 100% |
| Chrysene | 0.04 | 100% |
| Dibenzothiophene | 0.96 | 100% |
| Fluoranthene | 1.42 | 100% |
| Fluorene | 40.40 | 100% |
| Naphthalene | 169.43 | 100% |
| Phenanthrene | 20.39 | 100% |
| Pyrene | 0.90 | 100% |
| Retene | 0.90 | 100% |
| Triphenylene | 0.01 | 100% |
| 1-Methylphenanthrene | 0.11 | 94.12% |
| Benzo[a]pyrene | 0.03 | 94.12% |
| Benzo[k]fluoranthene | 0.01 | 94.12% |
| Benzo[a]fluorene | 0.04 | 88.24% |
| Cyclopenta[cd]pyrene | 0.05 | 88.24% |
| Benzo[j]fluoranthene | 0.02 | 82.35% |
| 1-Methylpyrene | 0.03 | 76.47% |
| 3,6-Dimethylphenanthrene | 0.21 | 76.47% |
| Benzo[b]fluorene | 0.02 | 64.71% |
| Benzo[c]fluorene | 0.02 | 64.71% |
| 1,8-Dimethylnaphthalene | 0.15 | 52.94% |
| Benzo[b]fluoranthene | 0.01 | 52.94% |
| Benzo[e]pyrene | 0.01 | 52.94% |

## Table S5. PAHs detected in WB samplers.

| **Chemical Name** | **Average Concentration (nmole/g WB)** | **Detection Frequency** |
| --- | --- | --- |
| 1-Methylnaphthalene | 0.07 | 100% |
| 2-Methylanthracene | 0.04 | 100% |
| 2-Methylnaphthalene | 0.15 | 100% |
| 2-Methylphenanthrene | 0.06 | 100% |
| Fluoranthene | 0.03 | 100% |
| Phenanthrene | 0.20 | 100% |
| Pyrene | 0.02 | 100% |
| Retene | 0.02 | 100% |
| Dibenzothiophene | 0.02 | 91.67% |
| Fluorene | 0.09 | 91.67% |
| Naphthalene | 0.07 | 91.67% |
| 2-Ethylnaphthalene | 0.02 | 66.67% |
| 1,6 and 1,3-Dimethylnaphthalene | 0.06 | 58.33% |
| 1,2-Dimethylnaphthalene | 0.01 | 50% |
| 2,6- Dimethylnaphthalene | 0.04 | 50% |
| Acenaphthene | 0.12 | 50% |
| 1-Methylpyrene | 0.00 | 25% |

Table S6. Creosote relevant stationary PAHs comparison with and without NE1 influential point. ns=not significant (p-value>0.05). Asterisks indicate adjusted p-values < 0.1(Bonferroni-Hochberg procedure), and italicized p-values indicate borderline significance (p-value between 0.1-0.2).

| **Creosote-Relevant PAHs (Stationary Samplers)** | **P-value (with NE1 influential point)** | **Adjusted p (with NE1 influential point)** | **P-value (without NE1 influential point)** | **Adjusted p (without NE1 influential point)** |
| --- | --- | --- | --- | --- |
| Acenaphthene | 0.0193 | 0.0734* | 0.0149 | *0.1791* |
| 2-Methylnaphthalene | 0.0554 | *0.1200* | 0.0623 (ns) | *0.1791* |
| Naphthalene | 0.0673 (ns) | *0.1346* | 0.1298 (ns) | 0.2596 |
| 1-Methylnaphthalene | 0.0404 | 0.0955* | 0.0416 | *0.1791* |
| Fluorene | 0.0150 | 0.0734* | 0.0131 | *0.1791* |
| 2,6-Dimethylnaphthalene | 0.0231 | 0.0734* | 0.0648 (ns) | *0.1791* |
| Phenanthrene | 0.0158 | 0.0734* | 0.0339 | *0.1791* |
| 1,6 and 1,3-Dimethylnaphthalene | 0.0214 | 0.0734* | 0.1108 (ns) | 0.2401 |
| 1,5-Dimethylnaphthalene | 0.0254 | 0.0734* | 0.0979 (ns) | 0.2314 |
| 1,4-Dimethylnaphthalene | 0.0164 | 0.0734* | 0.0524 (ns) | *0.1791* |
| Anthracene | 0.0231 | 0.0734* | 0.0689 (ns) | *0.1791* |
| Fluoranthene | 0.0371 | 0.0955* | 0.1575 (ns) | 0.2803 |
| Acenaphthylene | 0.4648 (ns) | 0.6676 | 0.0456 | *0.1791* |
| Dibenzothiophene | 0.0169 | 0.0734* | 0.0258 | *0.1791* |
| 2-Methylphenanthrene | 0.8079 (ns) | 0.8128 | 0.7983 (ns) | 0.7983 |
| Pyrene | 0.0836 (ns) | *0.1553* | 0.3256 (ns) | 0.5291 |
| Retene | 0.1960 (ns) | 0.3397 | 0.1617 (ns) | 0.2803 |
| Benz[a]anthracene | 0.5202 (ns) | 0.6676 | 0.7521 (ns) | 0.7866 |
| Benzo[a]fluorene | 0.4256 (ns) | 0.6509 | 0.7440 (ns) | 0.7866 |
| Chrysene | 0.5212 (ns) | 0.6676 | 0.7563 (ns) | 0.7866 |
| Benzo[a]pyrene | 0.6403 (ns) | 0.6937 | 0.6369 (ns) | 0.7866 |
| Benzo[b]fluorene | 0.5570 (ns) | 0.6676 | 0.7478 (ns) | 0.7866 |
| Benzo[ghi]perylene | 0.5906 (ns) | 0.6676 | 0.5404 (ns) | 0.7806 |
| Benzo[k]fluoranthene | 0.5774 (ns) | 0.6676 | 0.5746 (ns) | 0.7863 |
| Benzo[e]pyrene | 0.2935 | 0.4769 | 0.3627 (ns) | 0.5547 |
| Benzo[b]fluoranthene | 0.8128 (ns) | 0.8128 | 0.6166 (ns) | 0.7866 |

Table S7. Creosote relevant WB PAHs comparison with and without NE1 influential point. ns=not significant (p-value>0.05). Asterisks indicate adjusted p-values < 0.1(Bonferroni-Hochberg procedure), and italicized p-values indicate borderline significance (p-value between 0.1-0.2). N/a denotes creosote-relevant PAHs that were not detected in over 25% of WBs.

| **Creosote-Relevant PAHs (WB Samplers)** | **P-value (with NE1 influential point)** | **Adjusted p (with NE1 influential point)** | **P-value (without NE1 influential point)** | **Adjusted p (without NE1 influential point)** |
| --- | --- | --- | --- | --- |
| Acenaphthene | 0.0357 | 0.0774* | 0.0150 | 0.039* |
| 2-Methylnaphthalene | 0.0135 | 0.0439* | 0.0026 | 0.0085* |
| Naphthalene | <0.0001 | 0.0013* | 0.0005 | 0.0033* |
| 1-Methylnaphthalene | 0.0104 | 0.0439* | 0.0004 | 0.0033* |
| Fluorene | 0.1035 | 0.*1682* | 0.3889 (ns) | 0.6320 |
| 2,6-Dimethylnaphthalene | 0.1933 (ns) | 0.2474 | 0.6966 (ns) | 0.7672 |
| Phenanthrene | 0.1980 (ns) | 0.2474 | 0.7672 (ns) | 0.7672 |
| 1,6 and 1,3-Dimethylnaphthalene | 0.0338 | 0.0774* | 0.1694 (ns) | 0.3146 |
| 1,5-Dimethylnaphthalene | n/a | n/a | n/a | n/a |
| 1,4-Dimethylnaphthalene | n/a | n/a | n/a | n/a |
| Anthracene | n/a | n/a | n/a | n/a |
| Fluoranthene | 0.3787 (ns) | 0.4103 | 0.7625 (ns) | 0.7672 |
| Acenaphthylene | n/a | n/a | n/a | n/a |
| Dibenzothiophene | 0.0684 (ns) | 0.*1270* | 0.1328 (ns) | 0.2877 |
| 2-Methylphenanthrene | 0.0135 | 0.0439* | 0.0026 | 0.0085* |
| Pyrene | 0.2093 (ns) | 0.2474 | 0.5815 (ns) | 0.7560 |
| Retene | 0.4492 (ns) | 0.4492 | 0.4609 (ns) | 0.6657 |
| Benz[a]anthracene | n/a | n/a | n/a | n/a |
| Benzo[a]fluorene | n/a | n/a | n/a | n/a |
| Chrysene | n/a | n/a | n/a | n/a |
| Benzo[a]pyrene | n/a | n/a | n/a | n/a |
| Benzo[b]fluorene | n/a | n/a | n/a | n/a |
| Benzo[ghi]perylene | n/a | n/a | n/a | n/a |
| Benzo[k]fluoranthene | n/a | n/a | n/a | n/a |
| Benzo[e]pyrene | n/a | n/a | n/a | n/a |
| Benzo[b]fluoranthene | n/a | n/a | n/a | n/a |

Table S8. Hazard quotient and Cancer risk assessment for naphthalene. Hazard quotient = air concentration / reference concentration, cancer risk = concentration * unit risk. Values were compared to a prior study conducted in 2007 (Oregon Public Health Division, 2007)

| **Distance (m)** | **Hazard quotient** | **Exceeds 2007 study? (Y.N)** | **Cancer risk** | **Exceeds 2007 study? (Y.N)** |
| --- | --- | --- | --- | --- |
| **0-0.25** | 1.18E-01 | N | 1.20E-05 | N |
|  | 3.53E-02 | N | 3.60E-06 | N |
|  | 3.70E-02 | N | 3.77E-06 | N |
|  | 2.82E-01 | N | 2.88E-05 | N |
|  | 3.47E-02 | N | 3.54E-06 | N |
|  | 2.13E-02 | N | 2.17E-06 | N |
| **0.25-0.5** | 7.17E-02 | N | 7.31E-06 | N |
|  | 1.09E-01 | N | 1.11E-05 | N |
|  | 3.43E-02 | N | 3.50E-06 | N |
|  | 6.27E-02 | N | 6.39E-06 | N |
| **0.5-1.0** | 1.57E-02 | N | 1.60E-06 | N |
|  | 1.70E-02 | N | 1.73E-06 | N |
|  | 1.89E-02 | N | 1.93E-06 | N |
|  | 1.39E-02 | N | 1.41E-06 | N |
|  | 1.91E-02 | N | 1.95E-06 | N |
|  | 3.50E-02 | N | 3.57E-06 | N |

Table S9. Comparison of detection frequency between chemicals found in WBs and other studies using WB samplers. ^1^Anderson et al. (2017); ^2^Baum et al. (2020); ^3^Bergmann et al. (2017); ^4^Caban-^5^Martinez et al. (2020); ^6^De Vecchi et al. (2019); ^7^Dixon et al. (2018); ^8^Dixon et al. (2019); ^9^Doherty et al. (2020); Hendryx et al. (2020); Manzano et al. (2019); O Connell et al. (2014); Paulik et al. (2018); Reche et al. (2020); ^10^Rohlman et al. (2019A); ^11^Rohlman et al. (2019B); ^12^Romanak et al. (2019); ^13^Santiago et al. (2020); ^14^Wang et al. (2019), ^15^Wang et al. (2020). (Hamzai et al., 2022).

| **Chemicals with 100% Detection Frequency in WBs** | **Other Studies Reporting 100% Detection Frequency** | **Other Studies Reporting Chemical in ≥ 1 WB Sampler** |
| --- | --- | --- |
| 1-Methylnaphthalene | 4,12,14,15 | 1,2,3,5,6,7,8,9 |
| 2-Methylanthracene | 12 | 1,2,3,6,7,8,11,14 |
| 2-Methylnaphthalene | 4,12,14 | 1,2,3,6,7,8,10,11 |
| 2-Methylphenanthrene | 12,14 | 1,2,3,6,7,8,11,15 |
| Fluoranthene | 4,12 | 1,2,3,6,7,8,9,10,11,14,15 |
| Phenanthrene | 4,12,13,14,15 | 1,2,3,6,7,8,9,10,11 |
| Pyrene | 4,12 | 1,2,3,6,7,8,9,10,11,14,15 |
| Retene | 12 | 1,2,3,6,7,8,11,14,15 |

# FIGURES


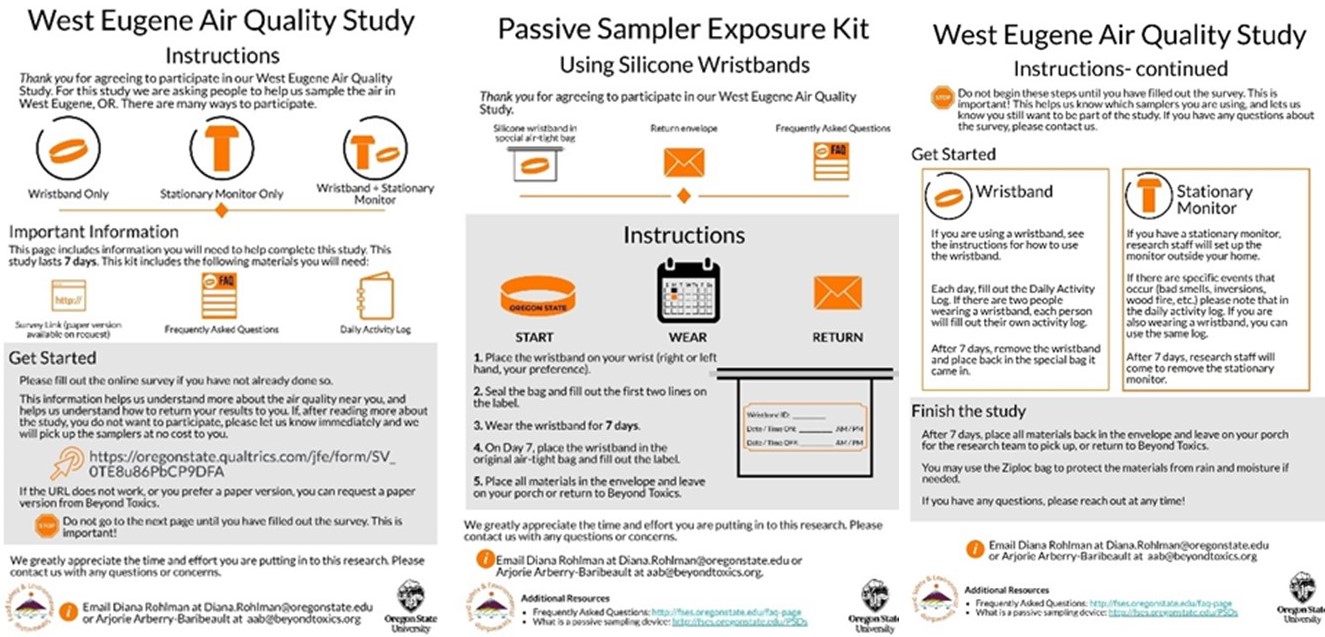
Figure S1. Instructional information regarding WB and stationary setup and use instructions. Instructions were disseminated to all study participants

## Figure S2. Final study design and layout of samplers in relation to the wood preservation facility.

**
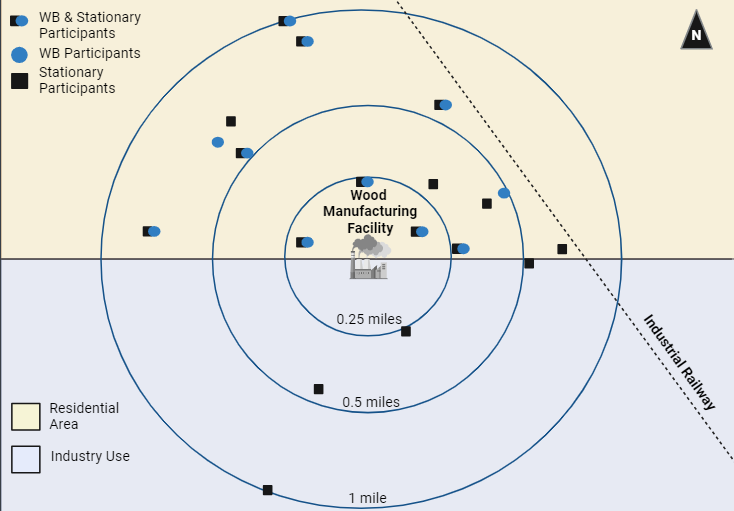
**

## Figure S3. Stationary sampler setup on residential participant property.

**
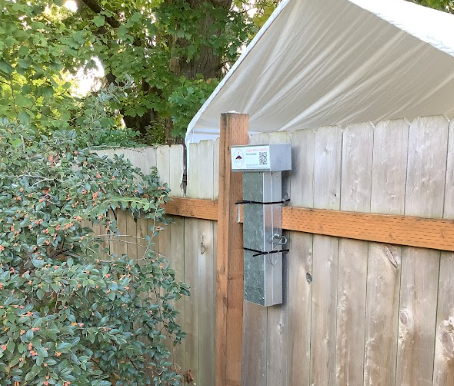
**

Figure S4. Pairwise distances between locations against differences in residuals for spatial analysis. (A) Pairwise distances for stationary samplers. (B) Pairwise distances for stationary samplers without influential point NE1. (C) Pairwise distances for WB samplers. (D) Pairwise distances for WB samplers without influential point NE1.

**
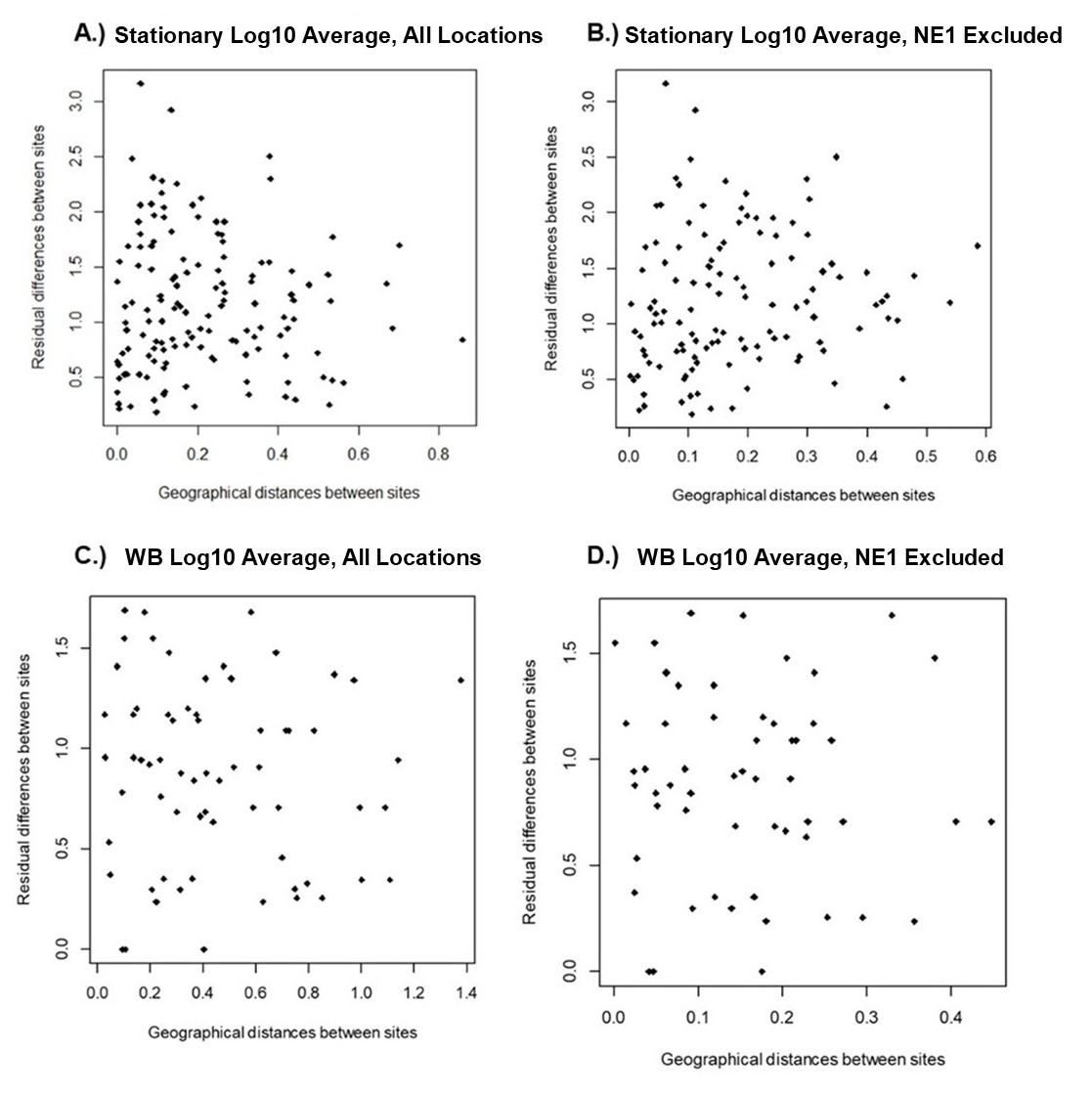
**

Figure S5. PAH detection in stationary samplers according to distance without the presence of influential sampler NE1. (A) PAHs detected in 25% or more of the samplers were included in this analysis. The average PAH concentration was calculated for each sampling ring (0.25, 0.5, and 1.0 miles). (B) Influential point NE1 was removed from regression and values were log transformed to further assess the relationship between PAH concentration and distance (p-value<0.0001, r2= 0.76).

**
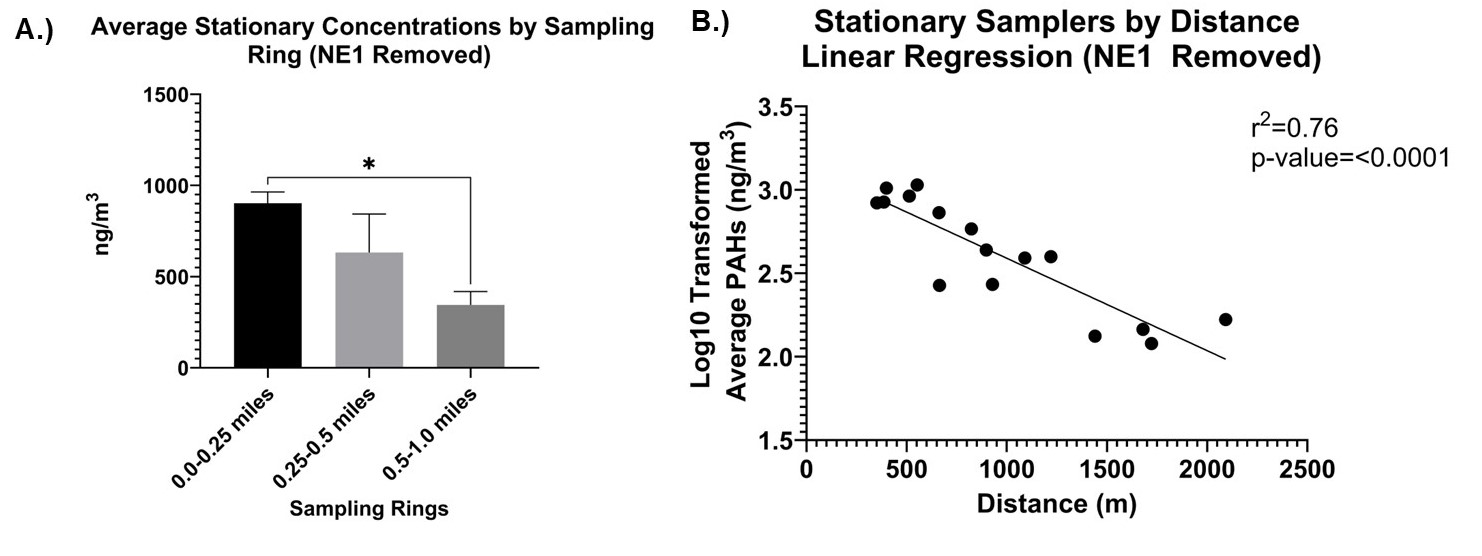
**

Figure S6. Creosote-relevant PAHs in samplers. (A) Stationary sampler PAHs. (B) WB sampler PAHs. Significance is noted for PAHs that showed significance between the inner and outer most sampling rings (one-way ANOVA, p-value<0.05).

**
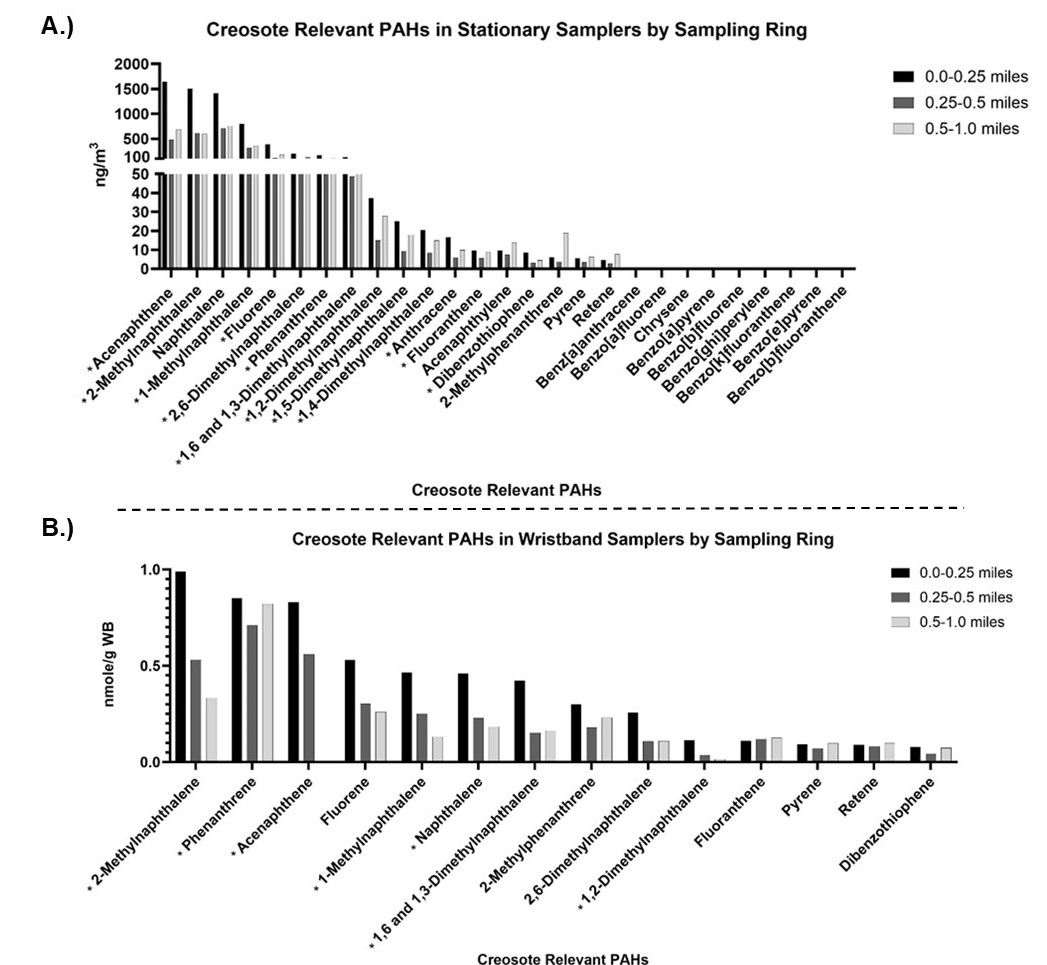
**

Figure S7. PAH detection in WB samplers according to distance without the presence of influential sampler NE1. PAHs detected in 25% or more of samplers were included in analysis. (A) Influential point NE1 was removed from the dataset prior to regression analysis and values were log transformed to further assess the relationship between PAH concentration and distance (p-value=0.0142, r2=0.51). (B) The average PAH concentration was calculated for each sampling ring (0.25, 0.5, 1.0 miles).


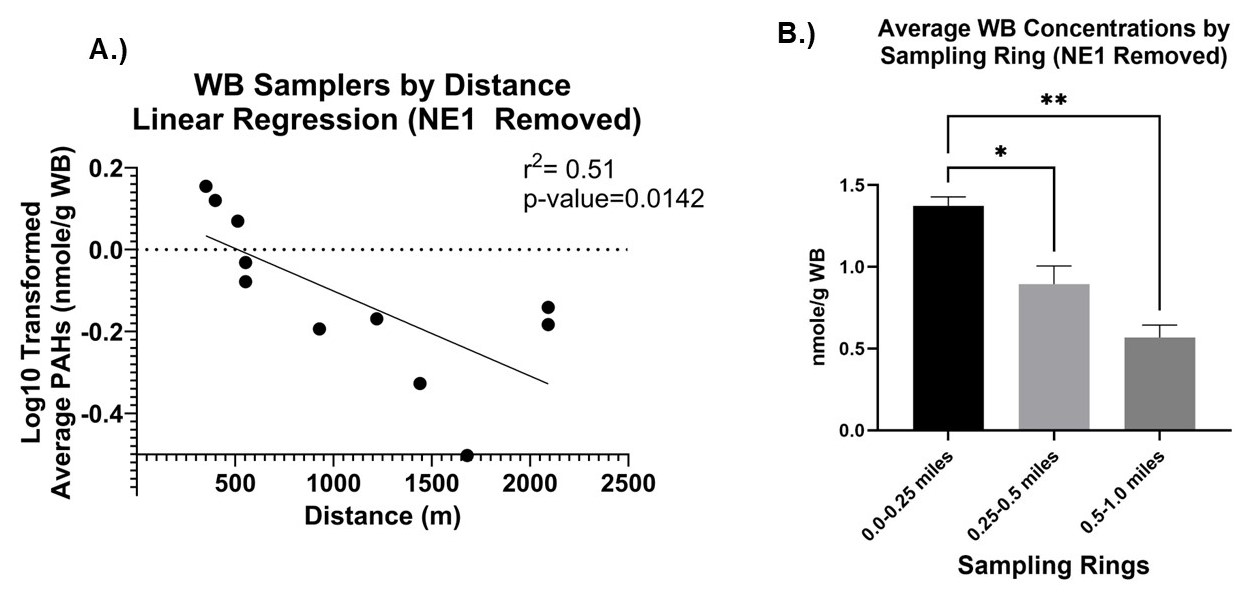


Figure S8. PAHs by sampling ring in stationary samplers. All PAHs were detected in over 25% of all stationary samplers. In cases where a PAH was not detected, the LOD/2 was input. *Asterisks indicate statistical significance between the inner and outer most sampling rings (one-way ANOVA, p-value <0.05).
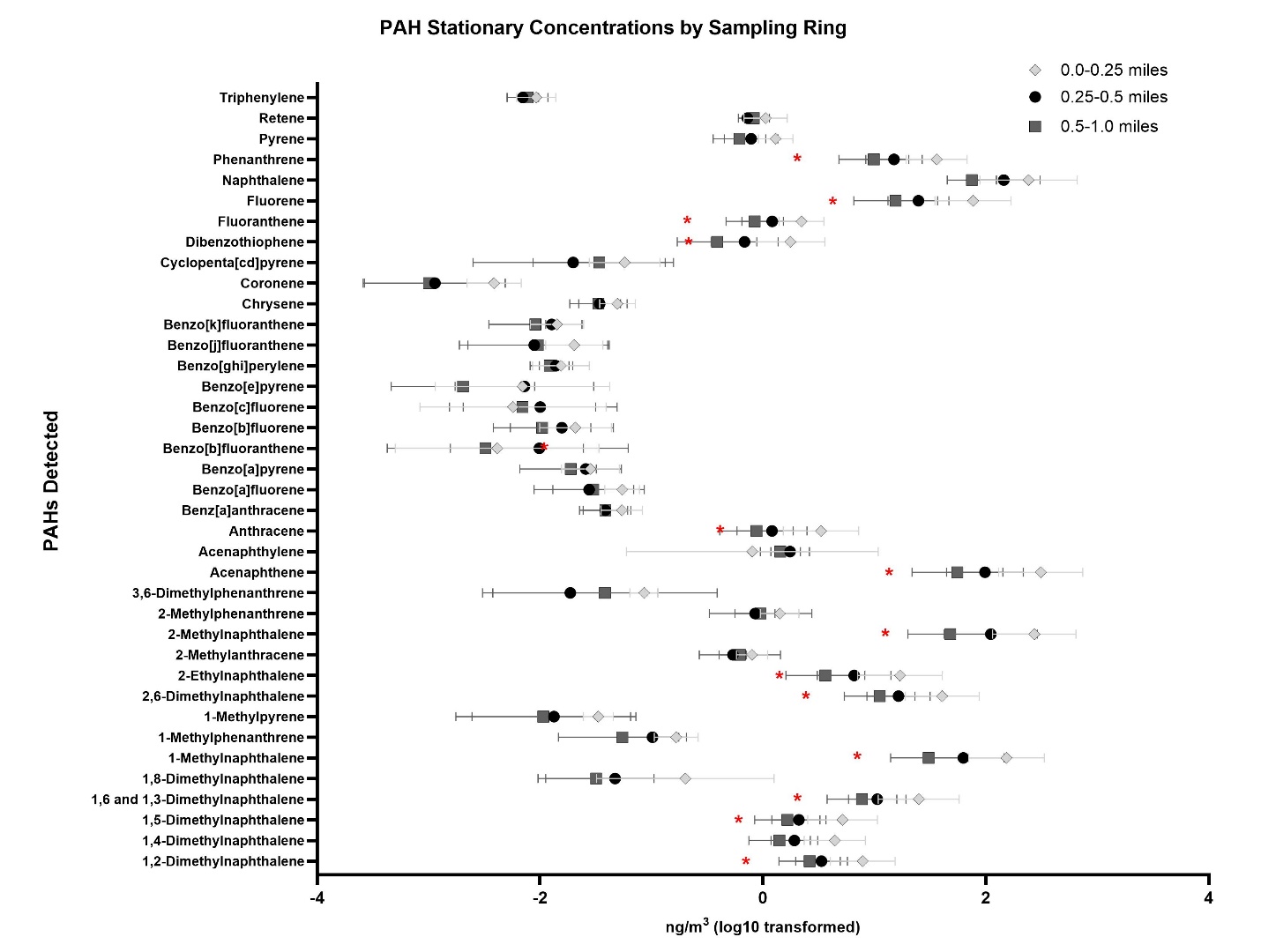


Figure S9. PAHs by sampling ring in WB samplers. All PAHs were detected in over 25% of all stationary samplers. In cases where a PAH was not detected, the LOD/2 was input. *Asterisks indicate statistical significance between the inner and outer most sampling rings (one-way ANOVA, p-value <0.05).
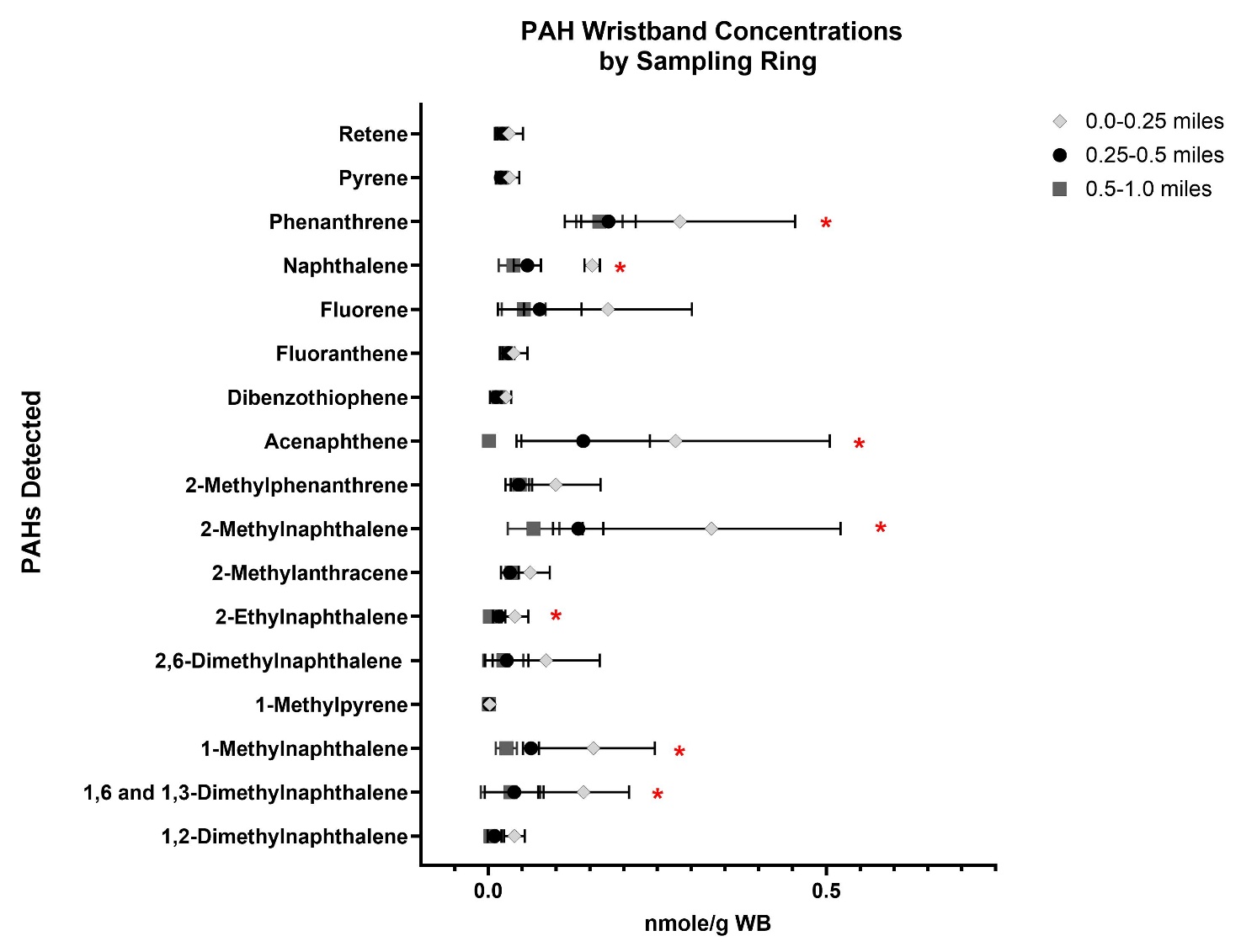


**
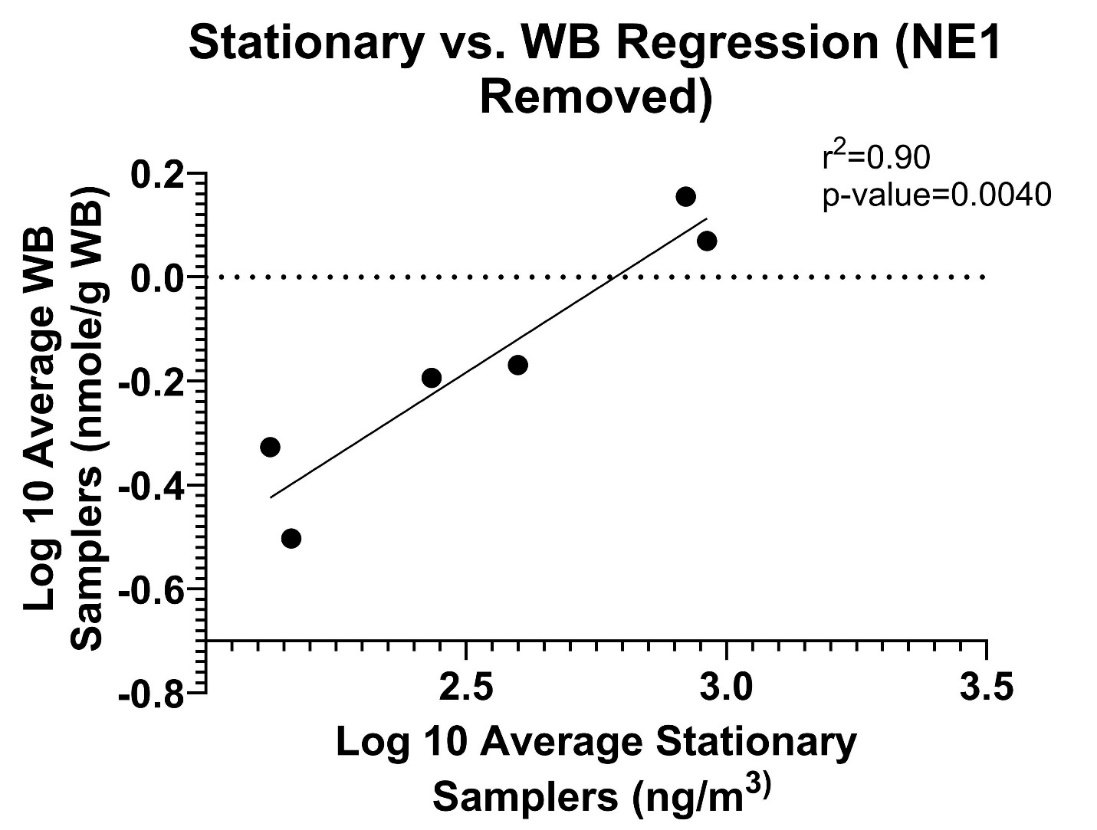
**Figure S10. Correlation between stationary and WB samplers with influential sampler NE1 removed. Values were log transformed to further assess the relationship between PAH concentration and distance (p-value<0.0040, r2= 0.90).

Figure S11. Percent of total sum PAH concentrations for stationary (A) and WB (B) samplers. PAHs detected in 25% or more of the samples were included in analysis.


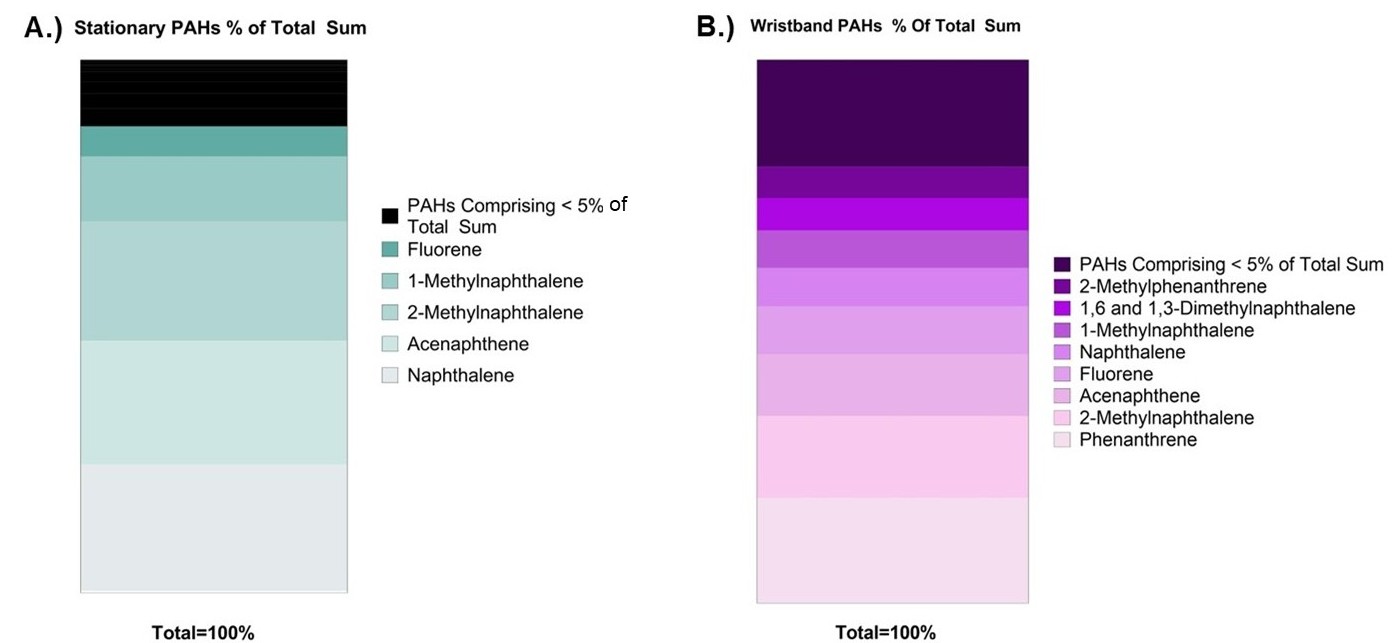


Figure S12. Average PAH concentrations according to sampling rings (0.25, 0.5, 1.0 miles) for participants identifying as smokers. Of the six participants who identified as smokers, one was excluded due to incomplete survey data. Among the remaining participants, three had only stationary samplers, while two had both stationary and wristband samplers. The figure shows the average PAH concentrations identified in stationary samplers only. Given the low number of wristband samplers associated with smokers, these samplers were excluded from analysis. Of the five participants, one was located in the NE, two were in the East, and the remaining two were in the South. PAHs included in analysis were detected in 25% or more of samplers (one-way ANOVA p-value <0.05), with no significant findings between sampling
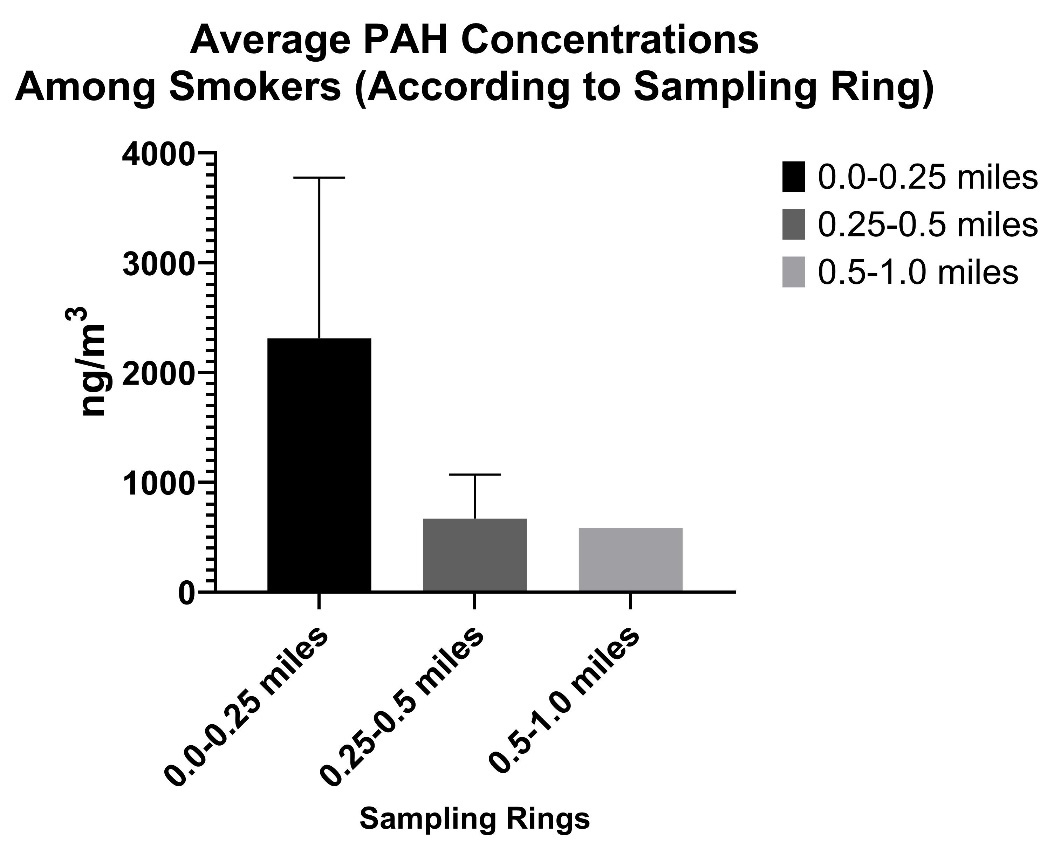
rings.

# APPENDICES

**Appendix A.** Community level report developed and disseminated to individual study participants, as well as the broader community in West Eugene. The report was disseminated by Beyond Toxics, and is available online: <https://ehsc.oregonstate.edu/our-research/research-highlights/west-eugene-air-quality-collaboration>

**Appendix B**. Anonymized individual report provided to all participants that received a stationary sampler.

**Appendix C**. Anonymized individual report provided to all participants that wore a wristband sampler.

*Note*: participants that received a stationary sampler and wore a wristband sampler received two reports, but these were sent as a combined PDF, in combination with the community level report.

Appendix A. Community-level report - 4 pages
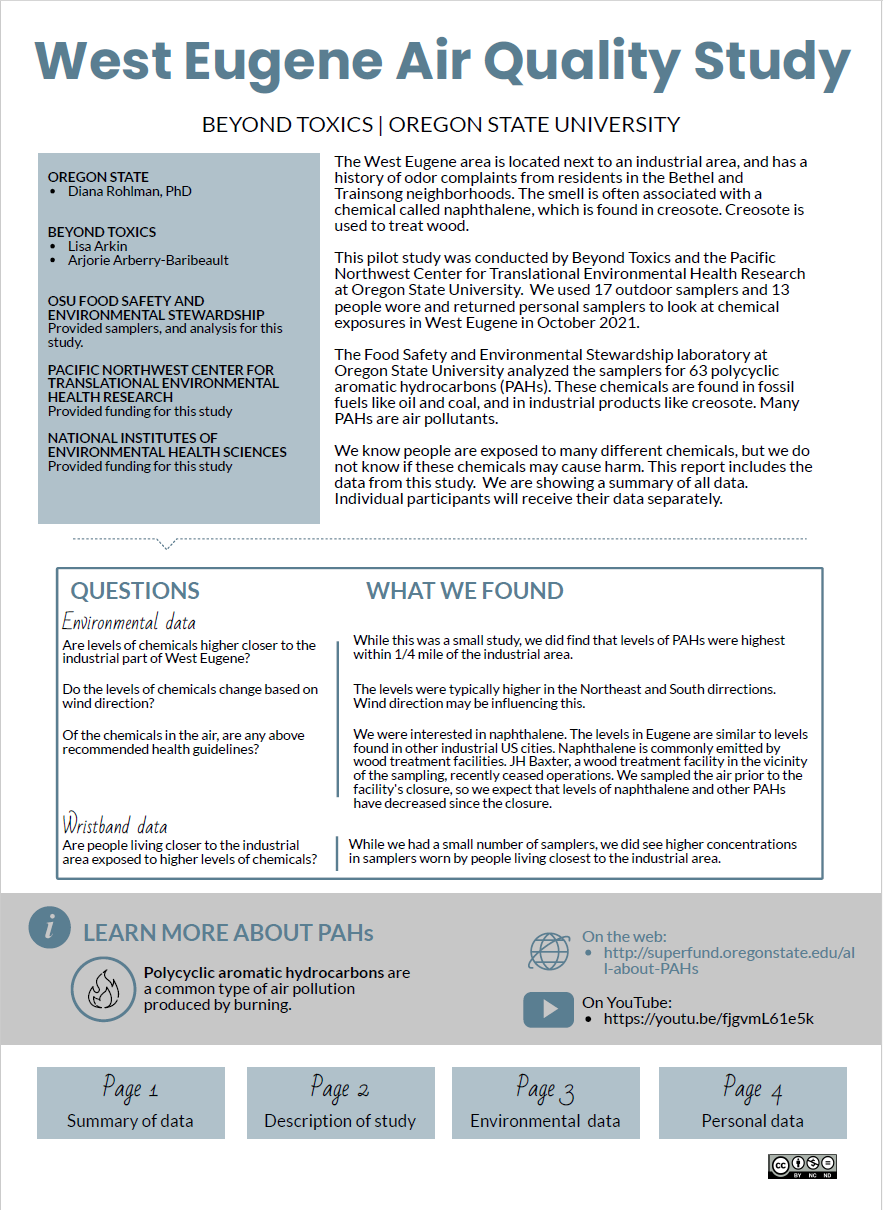


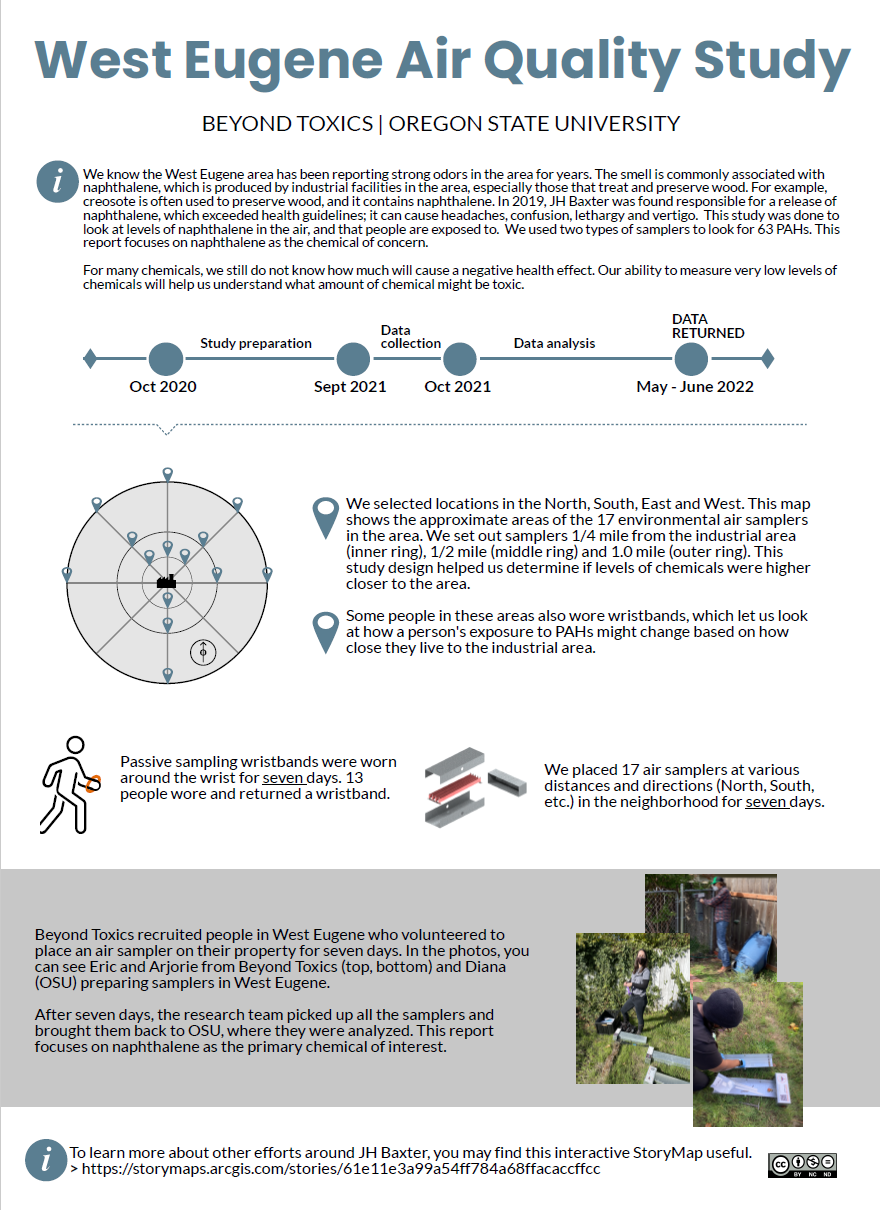


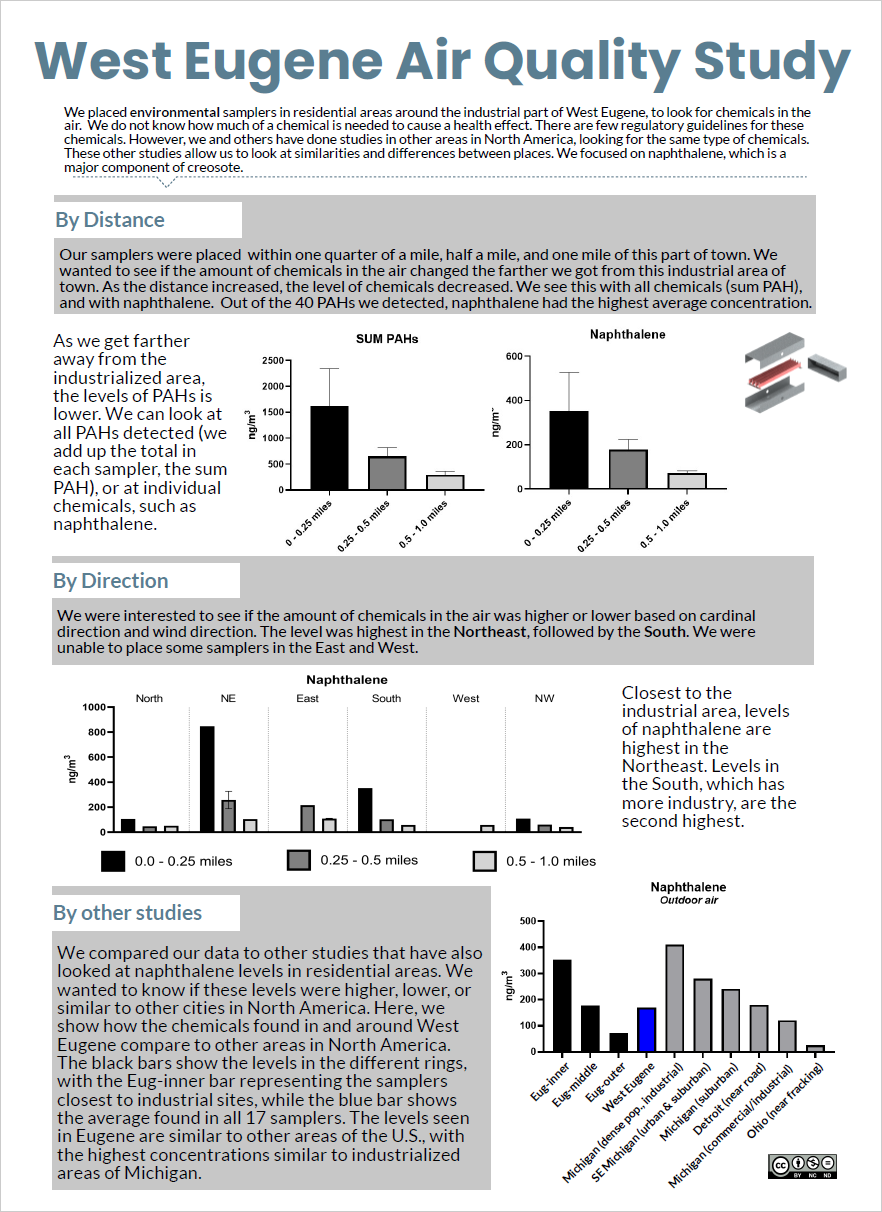


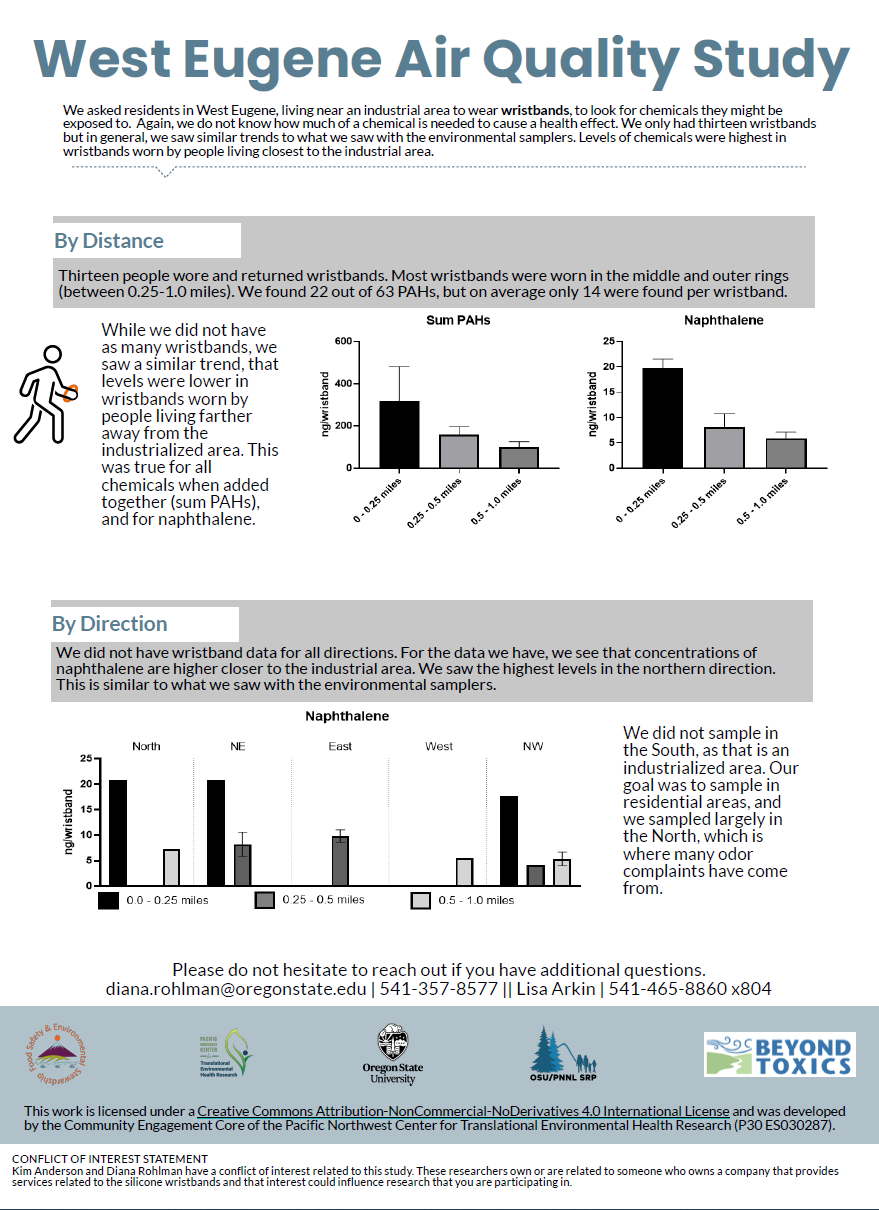


Appendix B. Anonymized individual stationary sampler report – 7 pages.


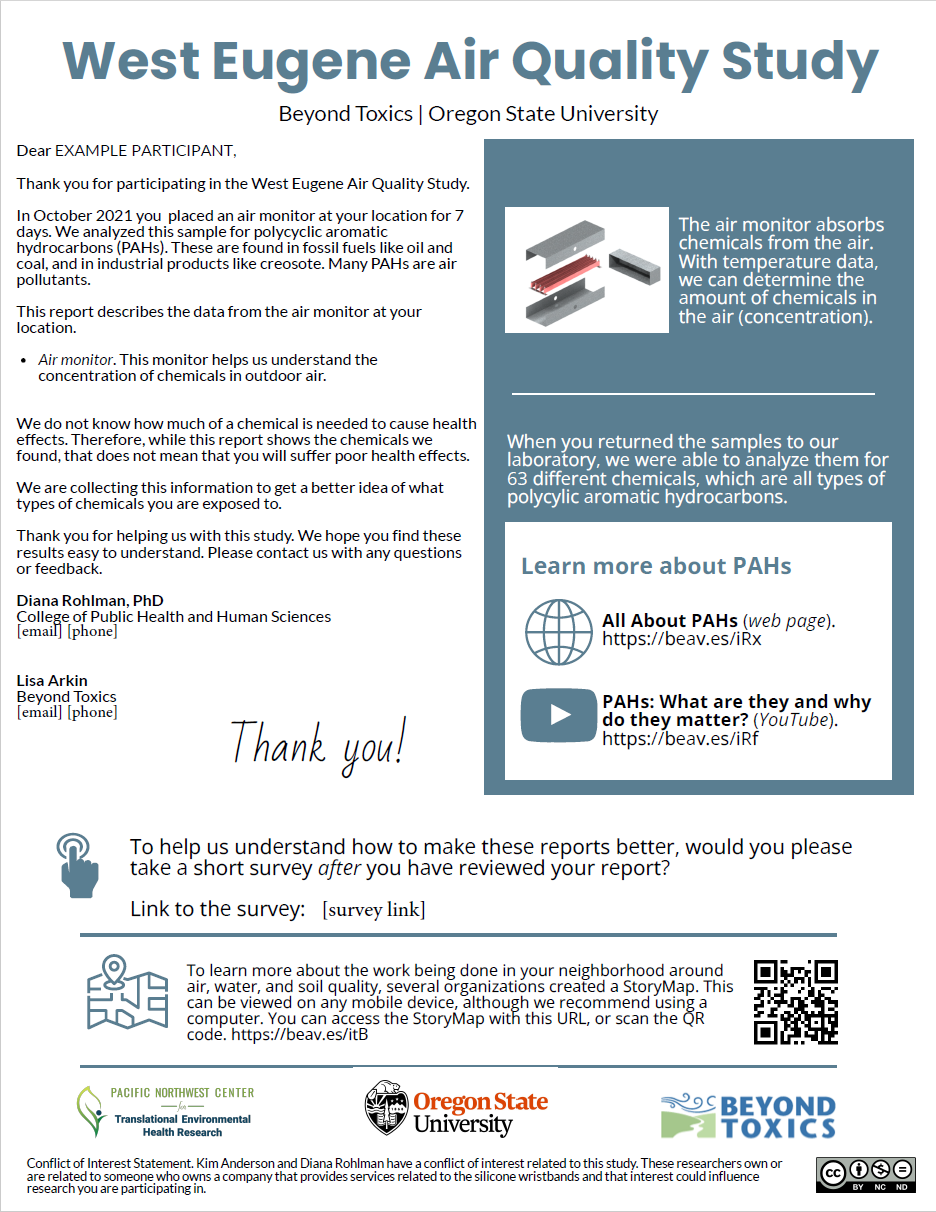


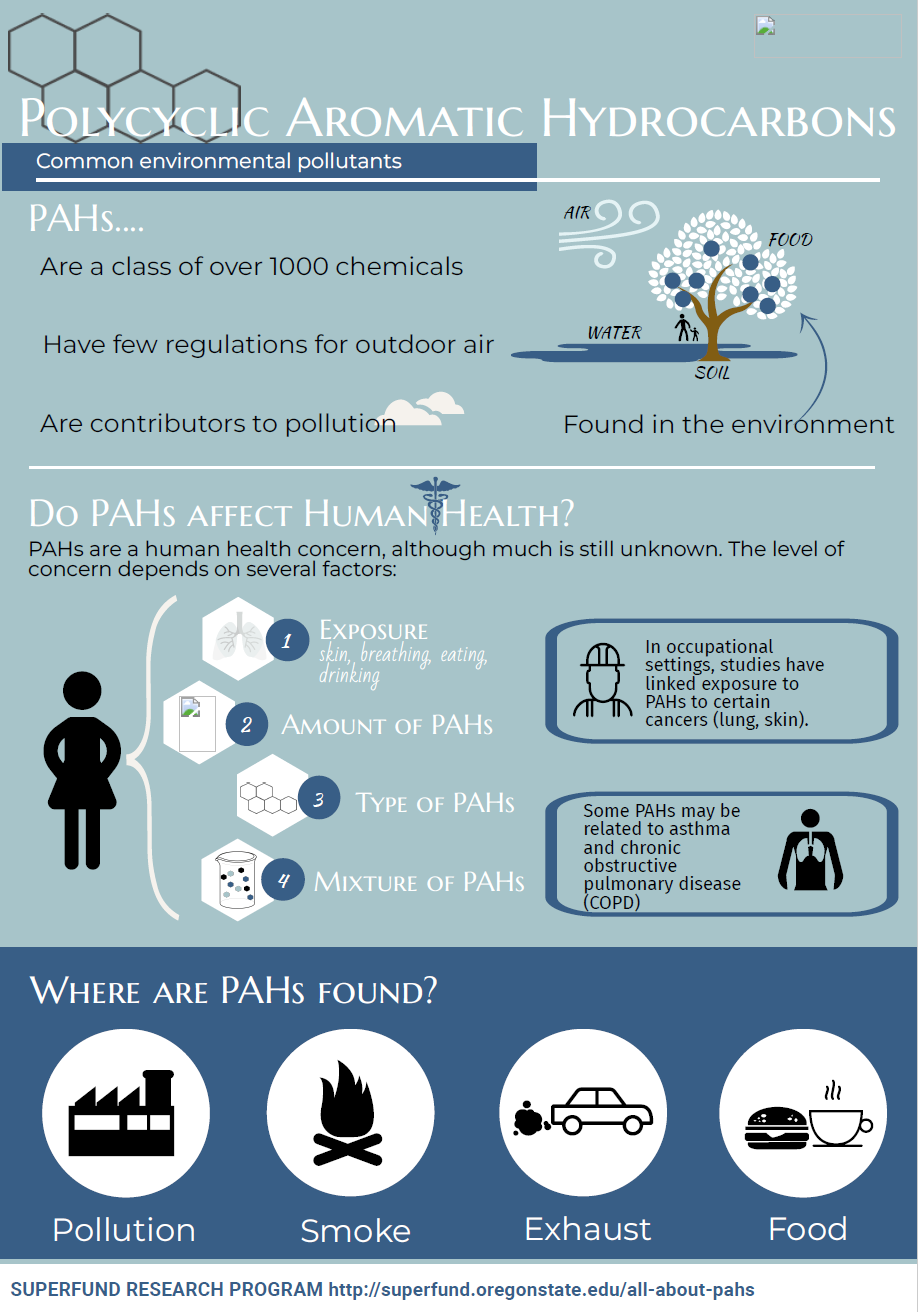


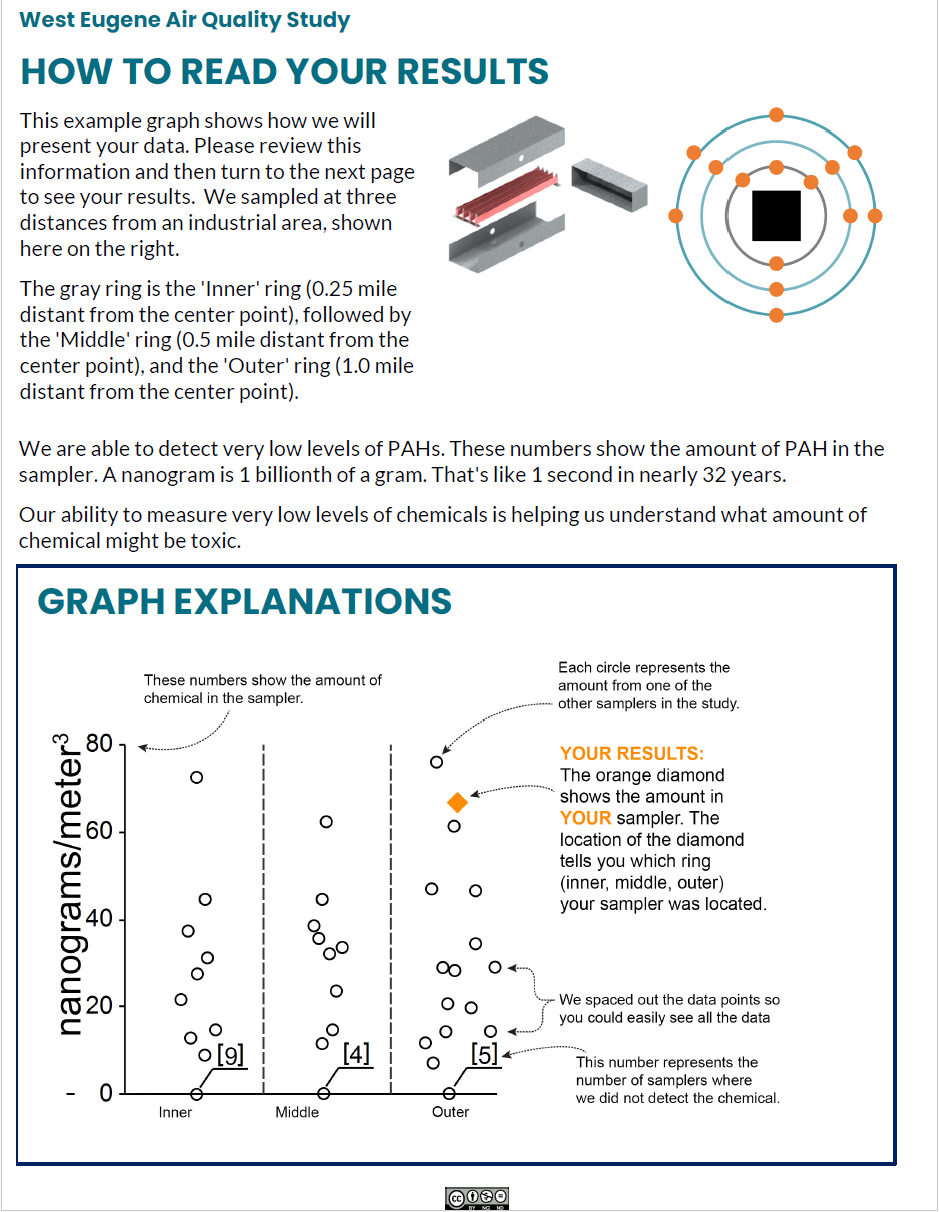


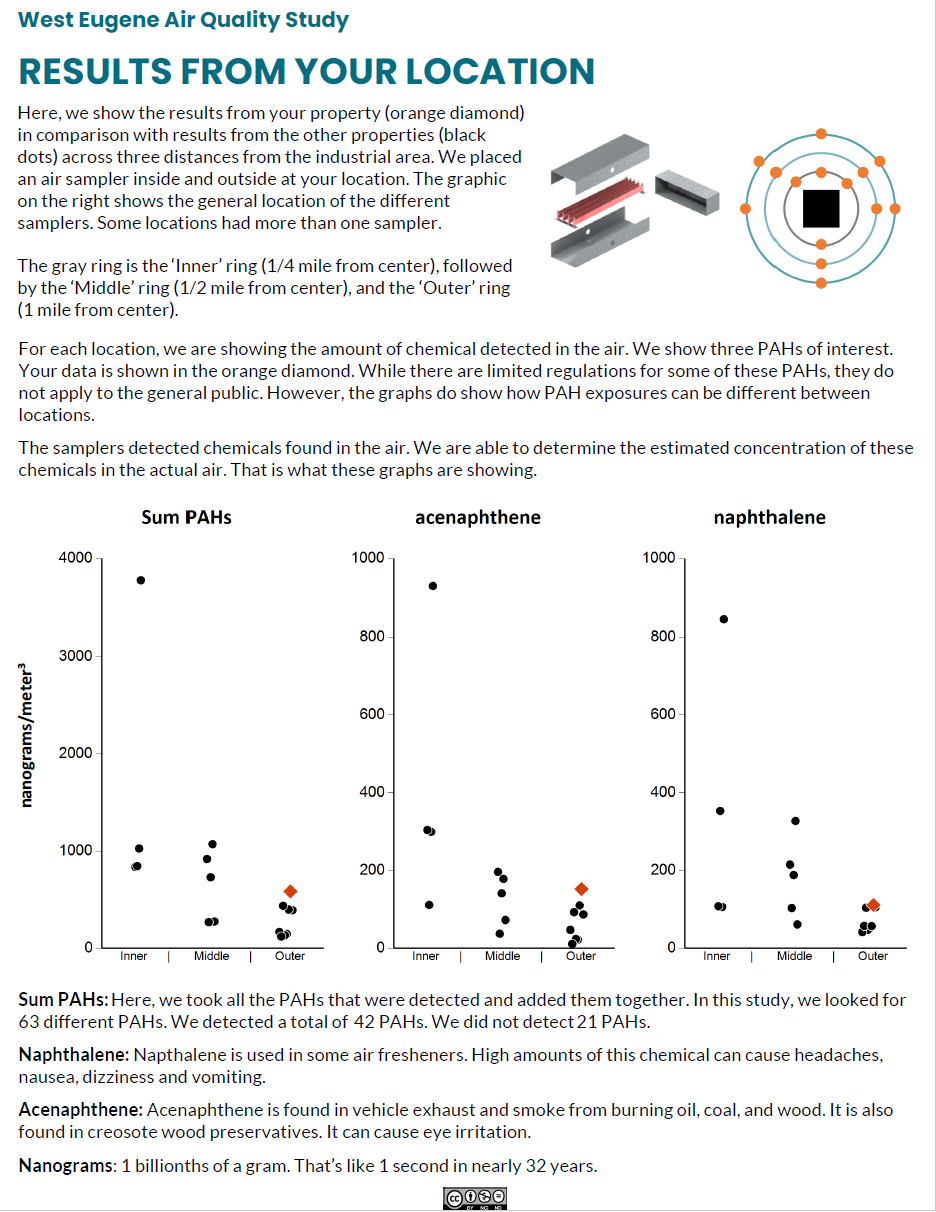


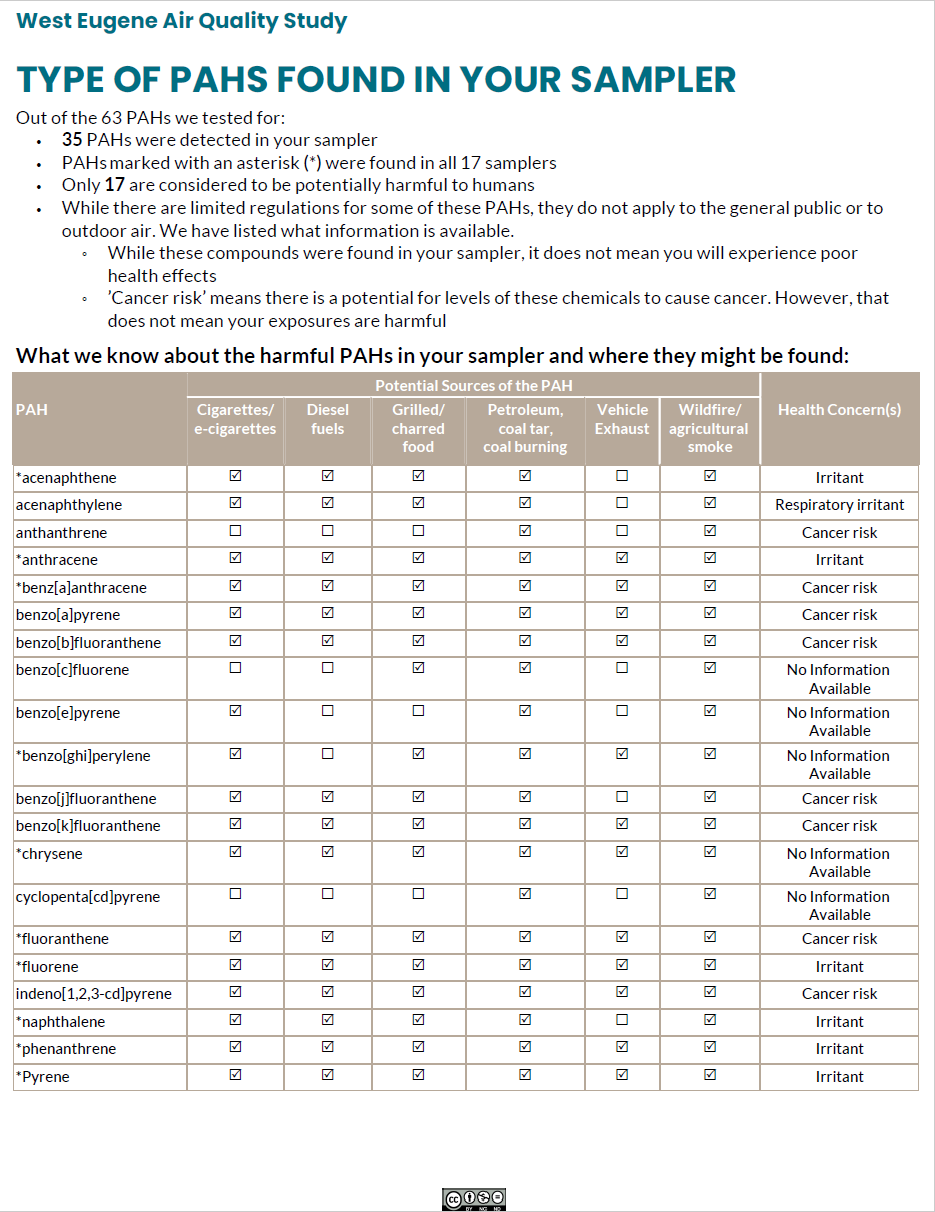


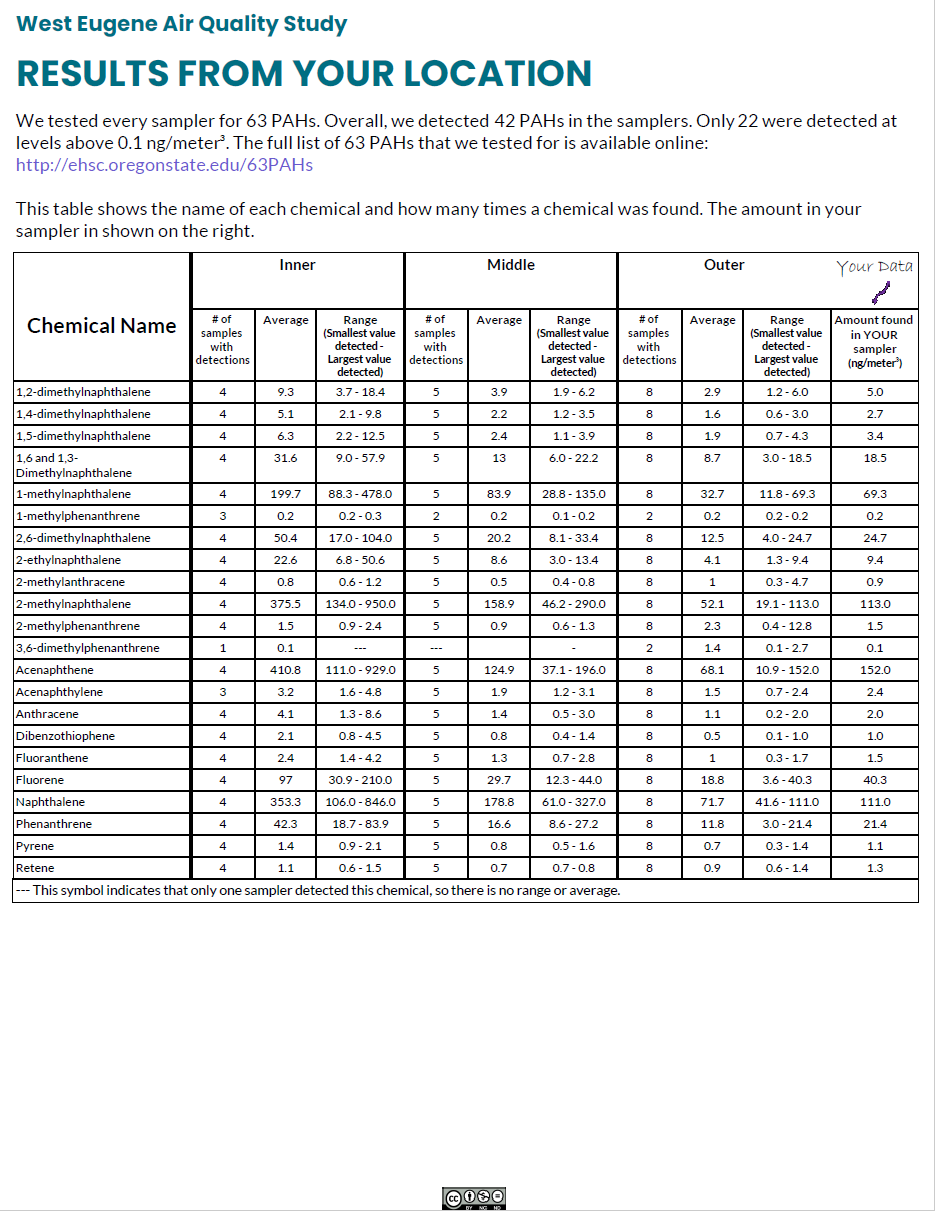


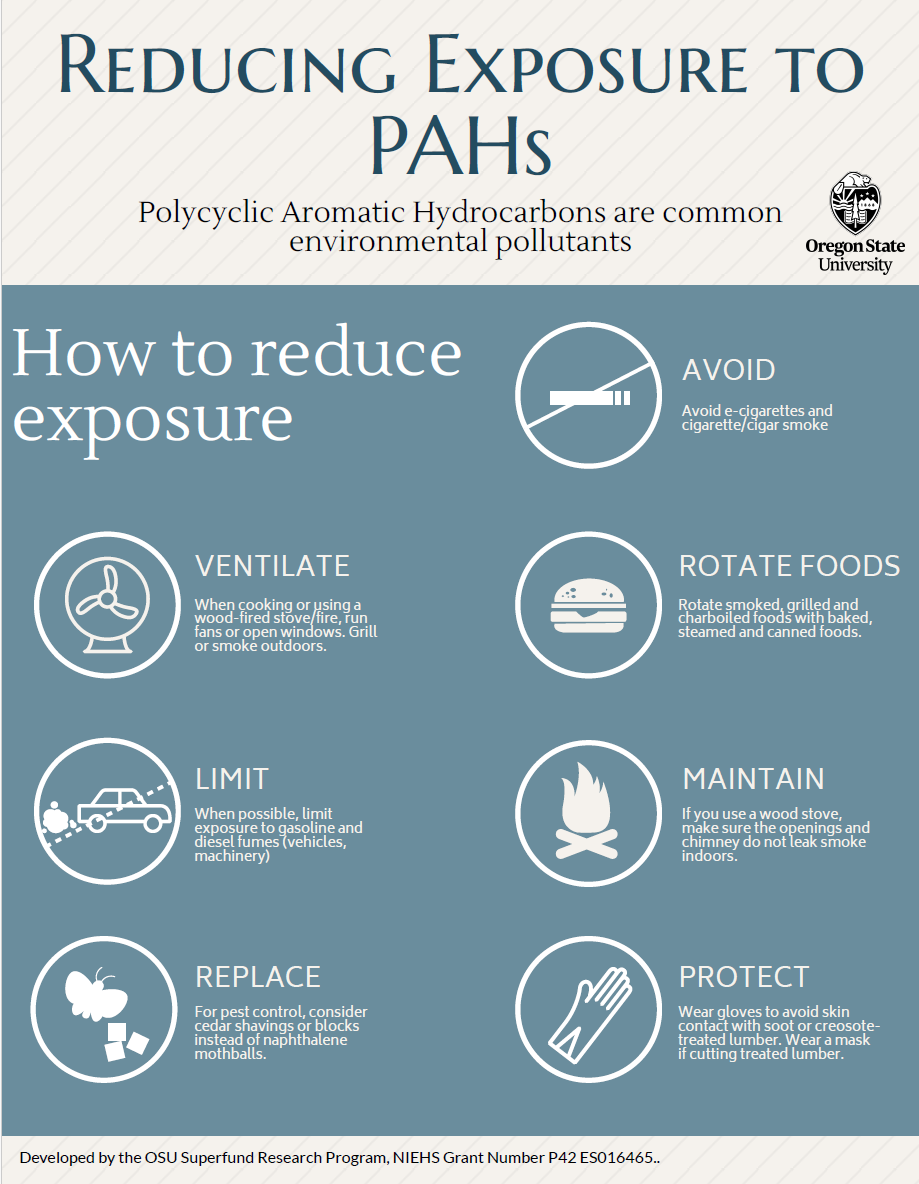


Appendix C. Anonymized individual wristband sampler report – 7 pages.


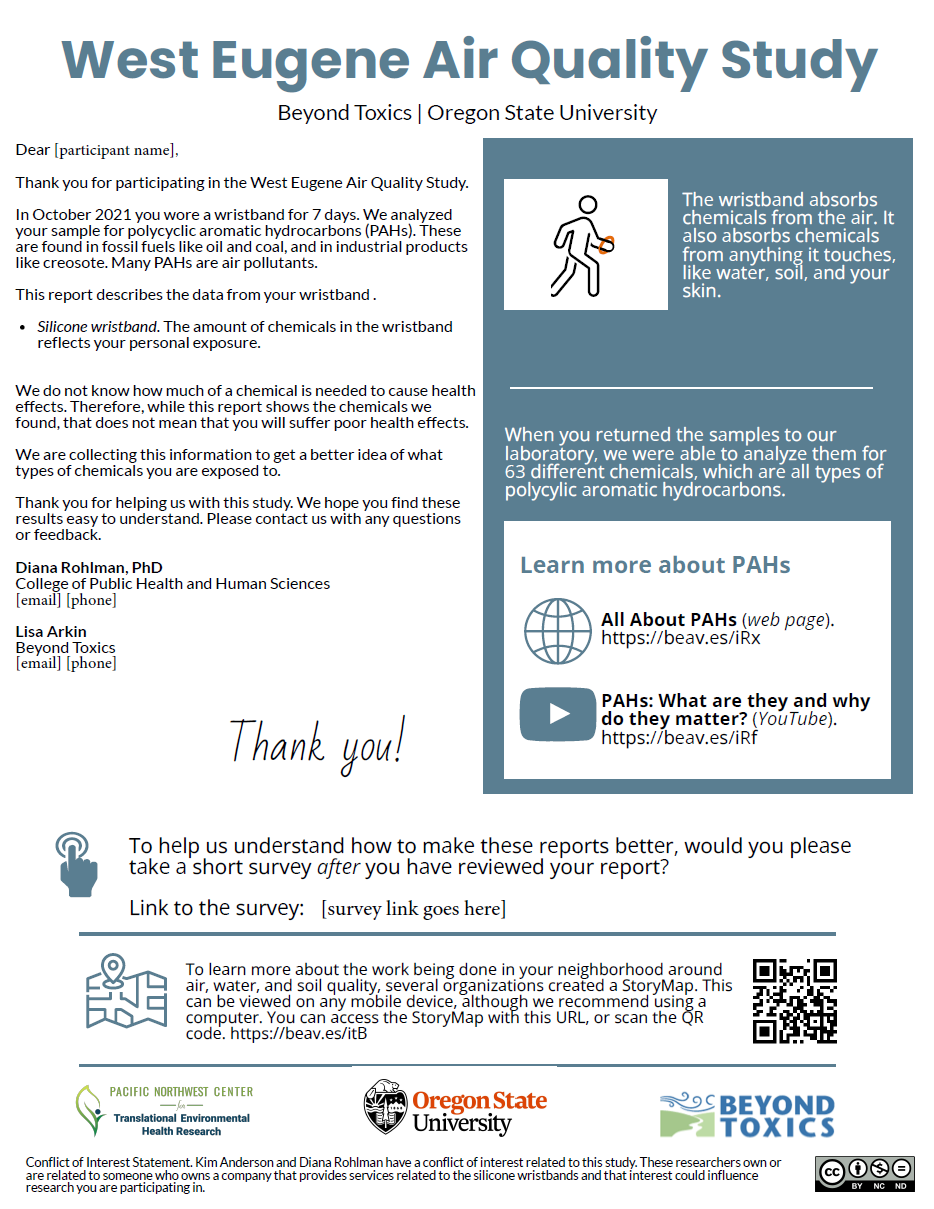


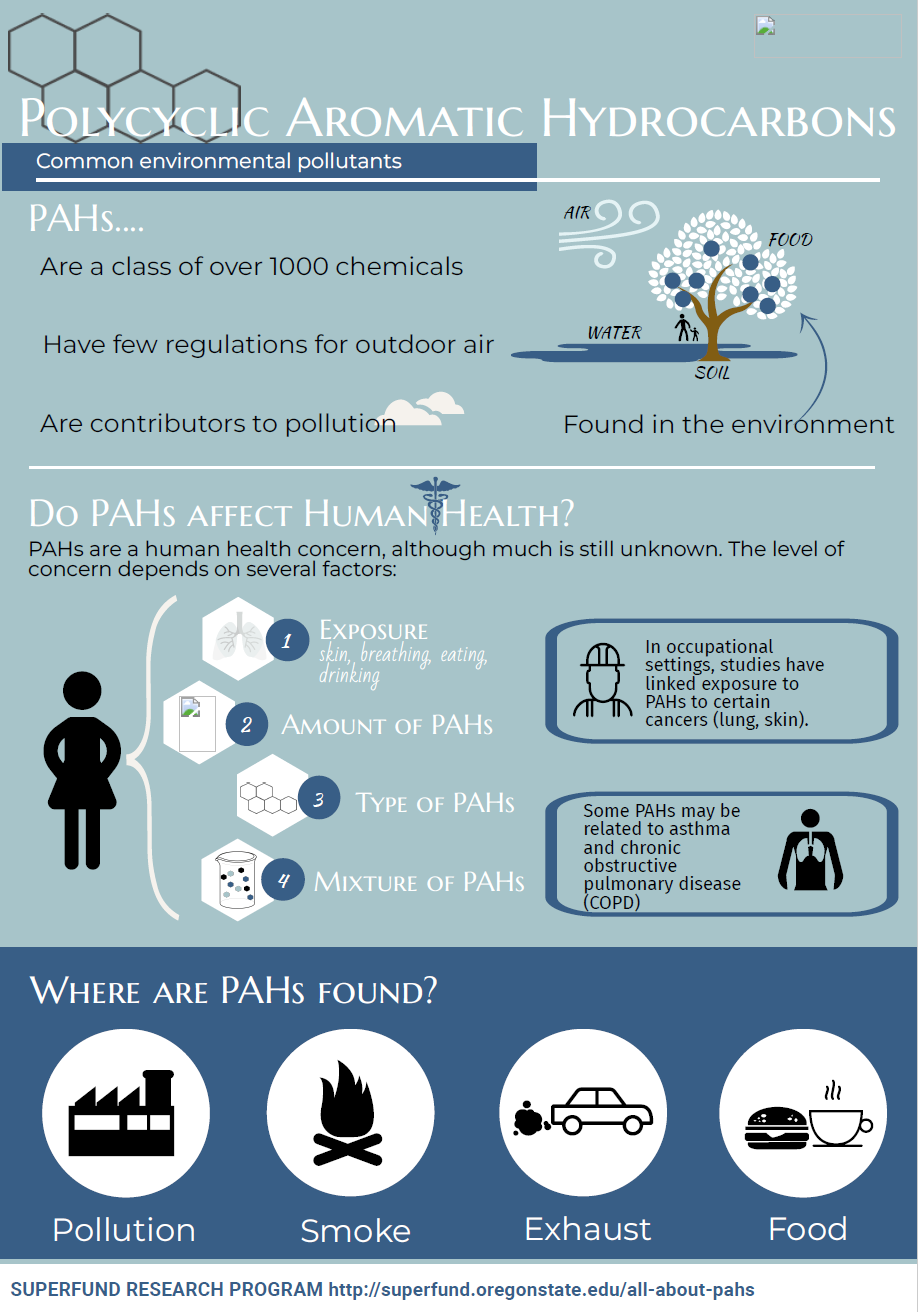


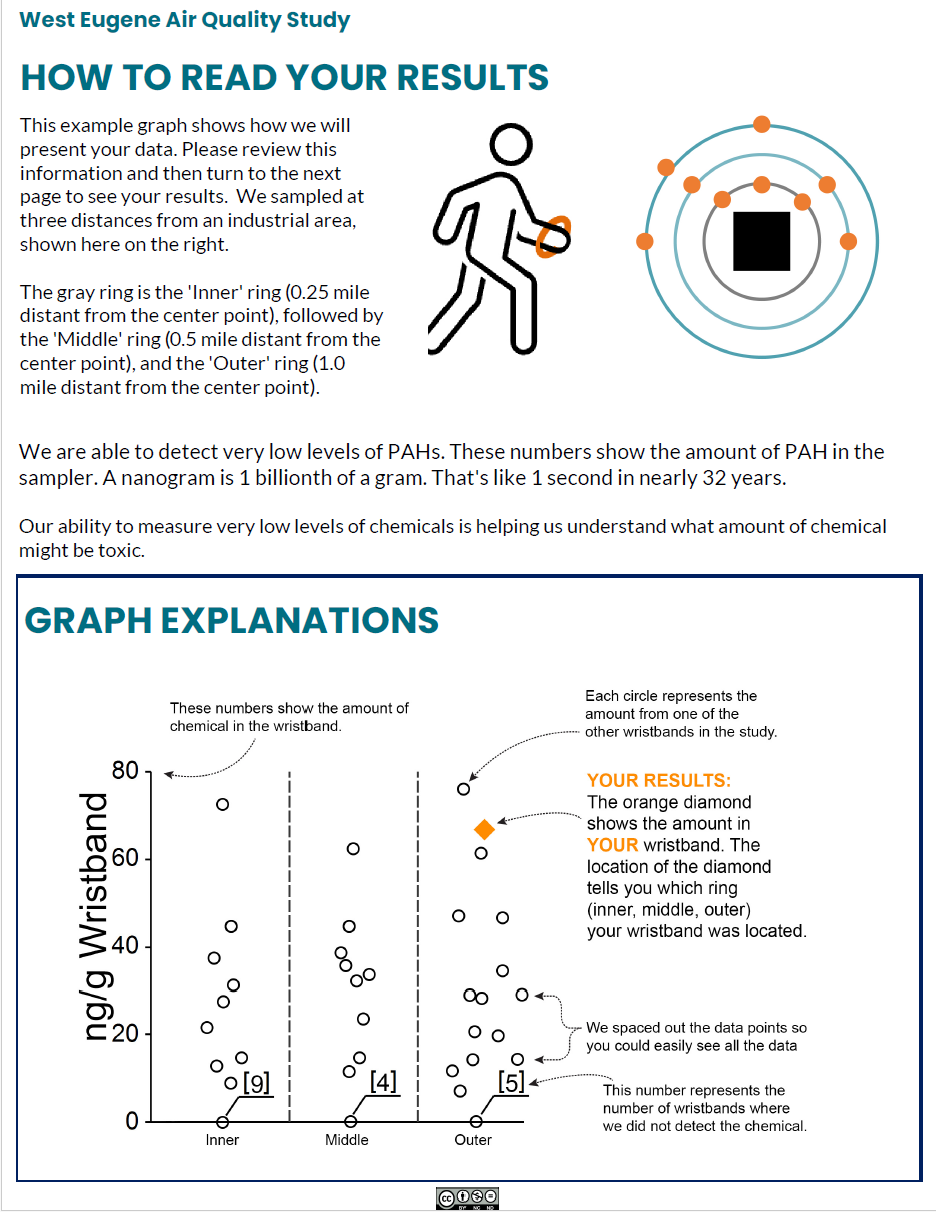


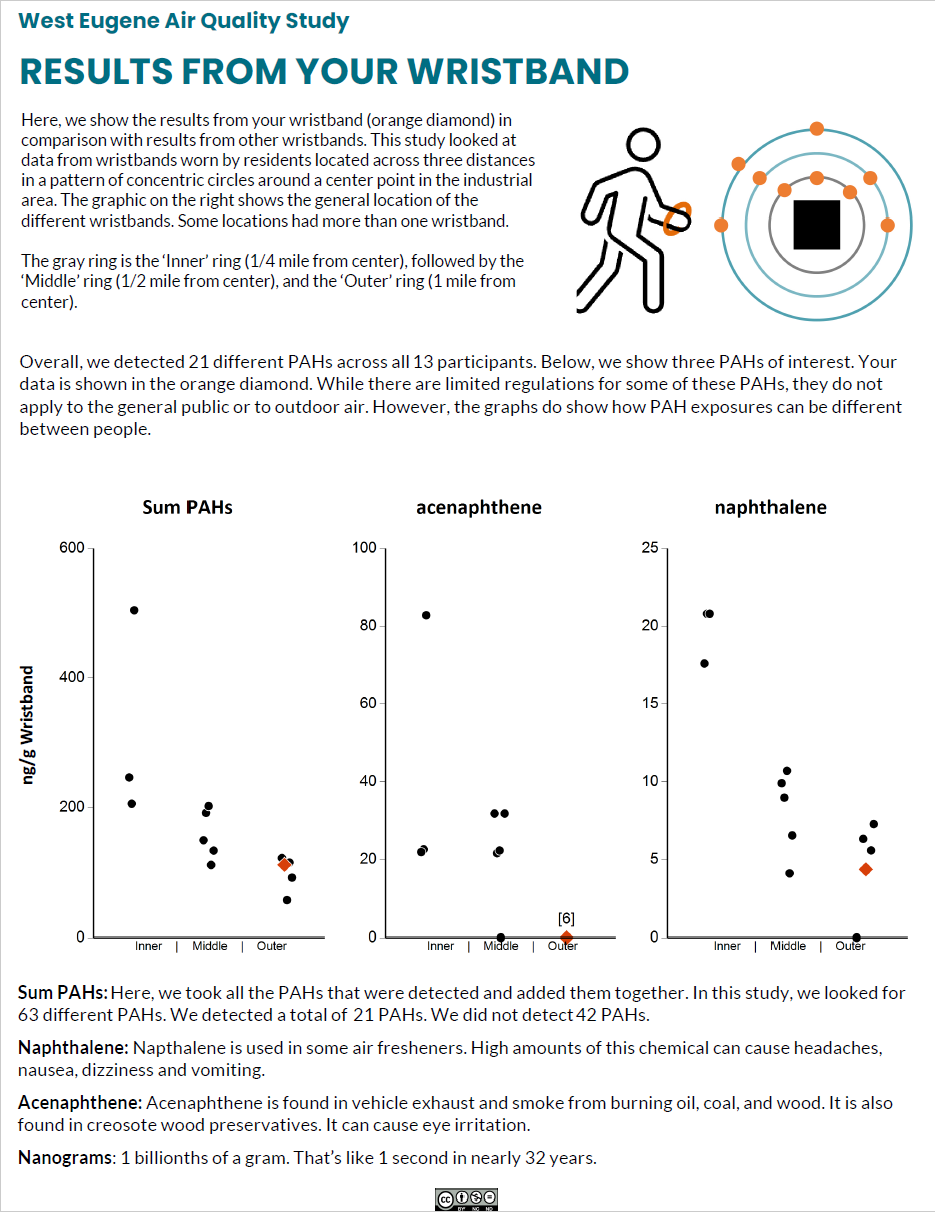


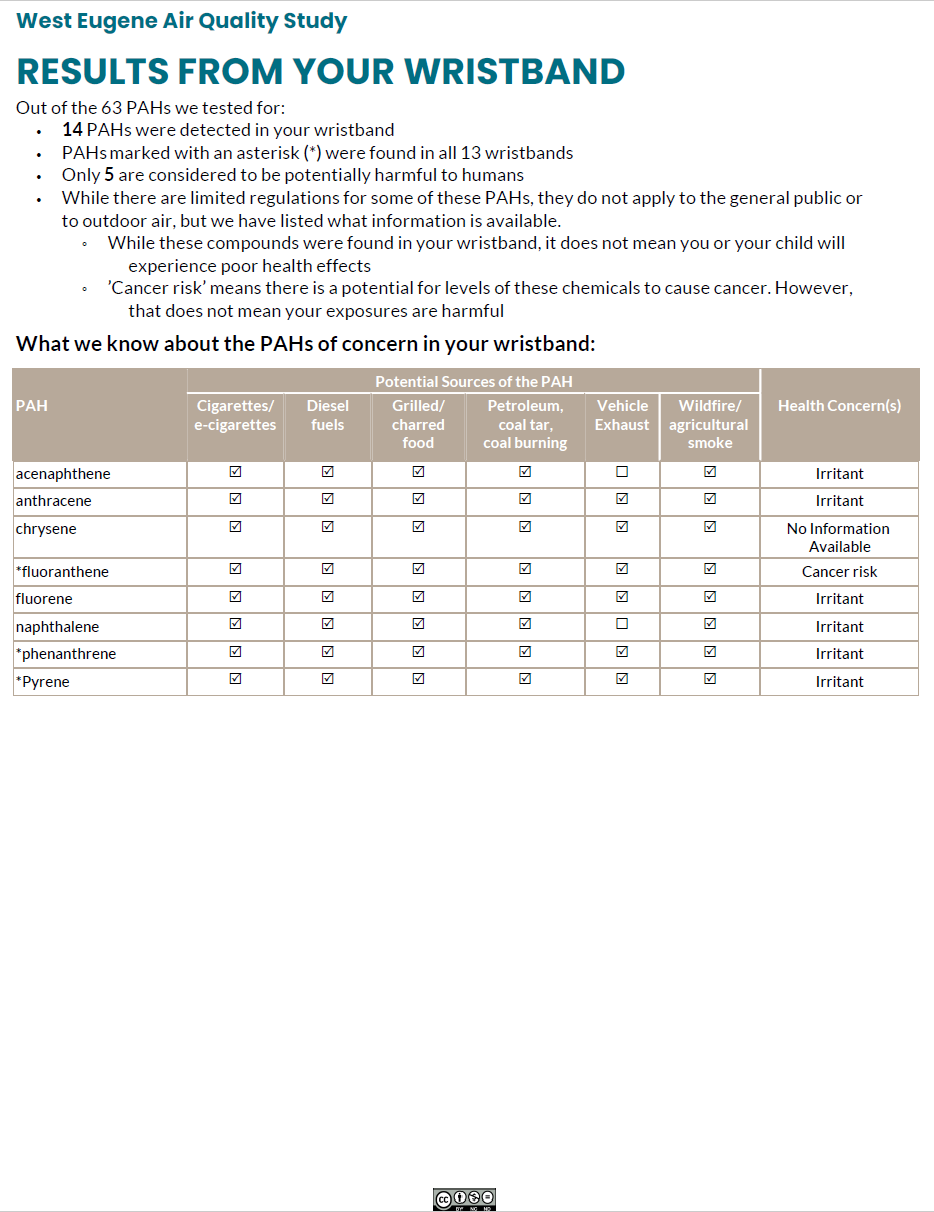


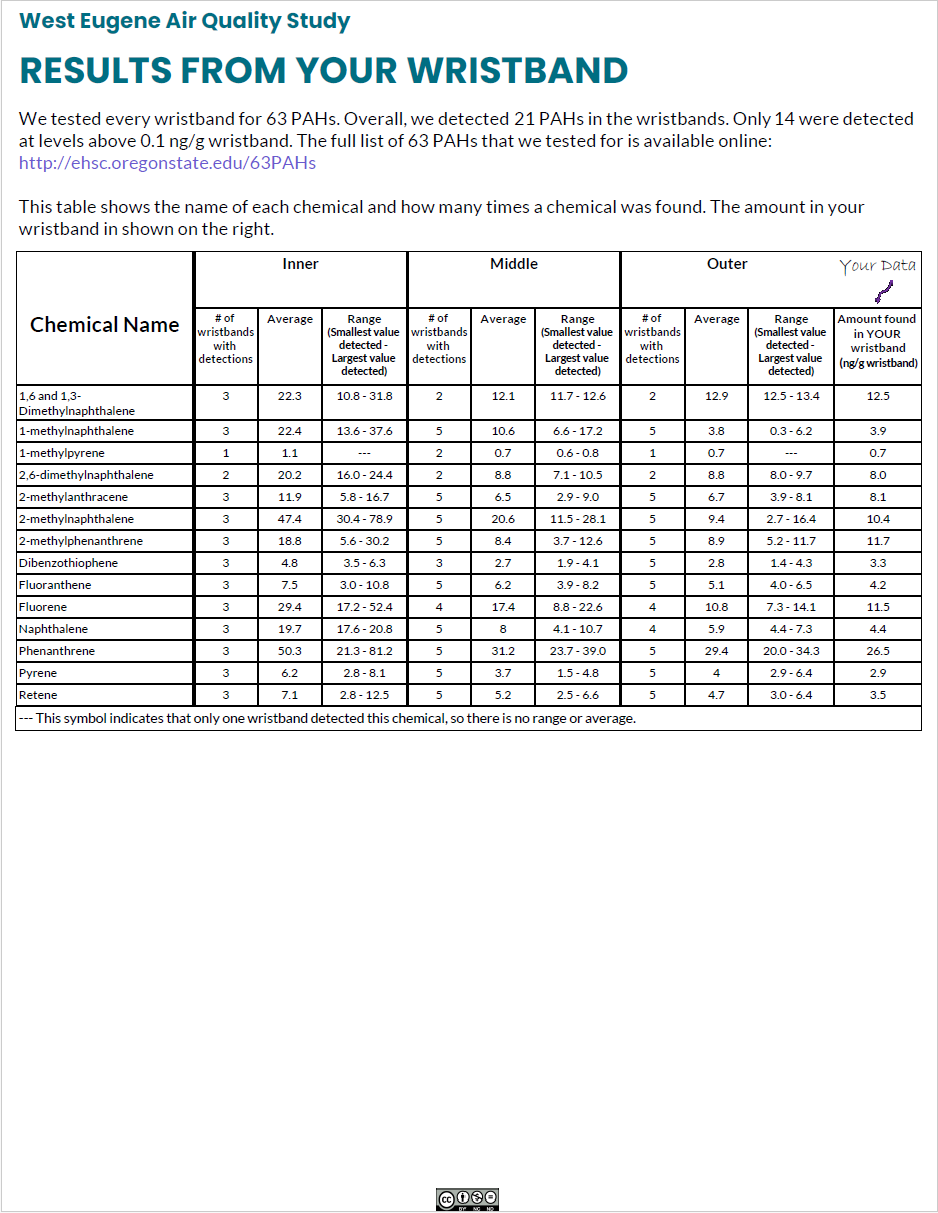


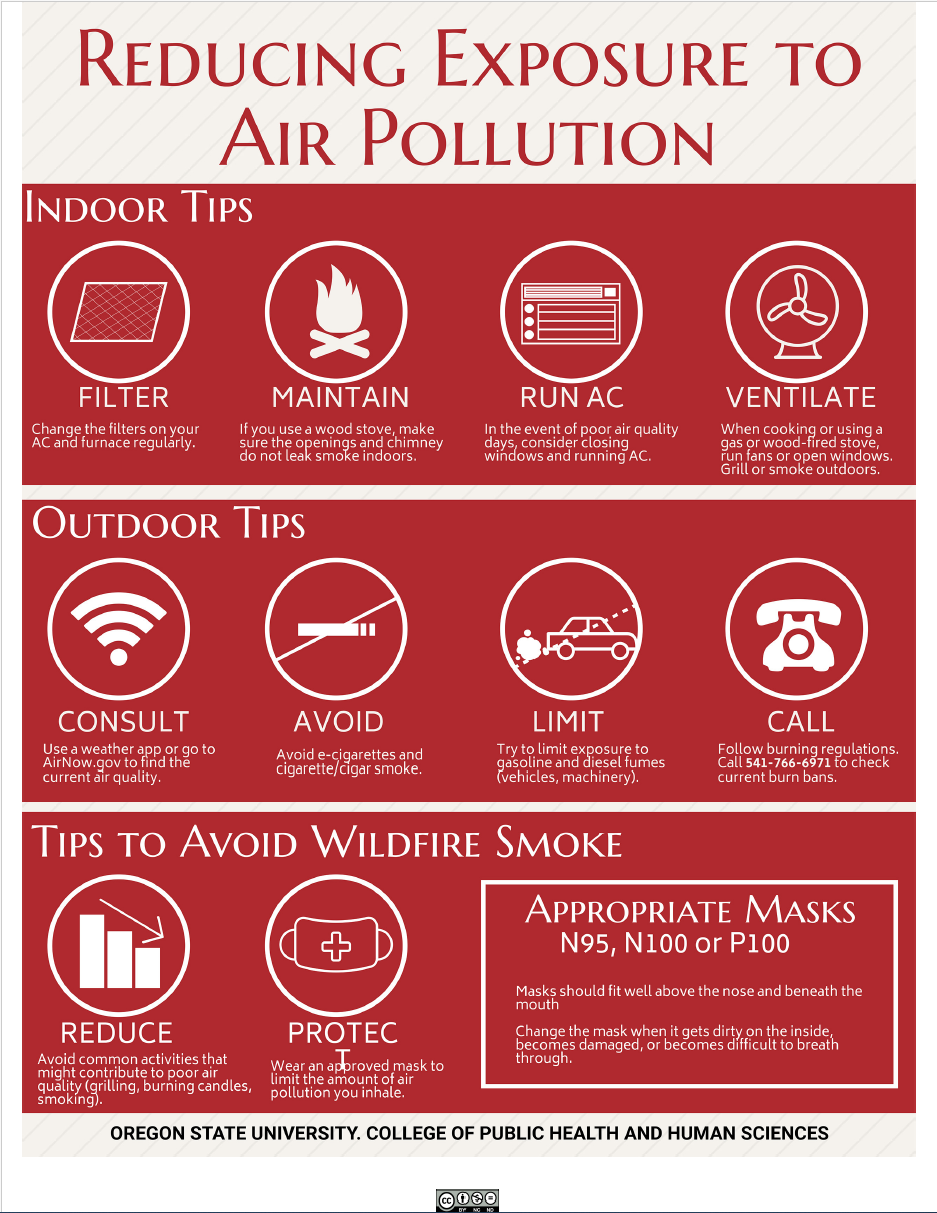


# CALCULATIONS

Time-weighted average concentrations for the vapor phase are determined using an empirical uptake model as described by Huckins et al.^1^

The air vapor-phase C_a_ (ng/m^3^) is calculated using the following equation:

$C_{a}=\frac{N_{analyte}}{V_{s}K_{sa(T)}(1-e^{\left( -\frac{R_{s}E}{V_{s}K_{sa(T)}} \right)})}$ Eq. S1

where N_analyte_ (ng) is the mass of the target compound in the sampler, V_s_ (cm^3^) is the volume of the sampler, K_sw_ is the sampler-water partition coefficient, K_sa(T)_ is the temperature corrected sampler-air partitioning coefficient, R_s_ is the PRC-derived sampling rate (m^3^/day), and E is the exposure duration (days).

Values for K*_sw_* are calculated according to the following empirical relationship reported by Lohmann 2012 using individual octanol-water partition coefficients (K*_ow_*)^1,2^:

$LogK_{sw}=1.22\left( logK_{ow} \right)-1.22$ Eq. S2

K_sa(298)_ is derived by first calculating the sampler-water partition coefficient (Eq. S2). K_sa(298)_ is then calculated using K_sw_ and the unitless Henry’s law constant (H’_298_) at 298 K as described by Lohmann^3^:

$K_{sa(298)}= \frac{K_{sw}}{{H'}_{298}}$ Eq. S3

The sampler-air partitioning coefficient is then temperature corrected as K_sa(T)_ using the Van’t Hoff equation^4-6^:

$K_{sa(T)}= K_{sa}* {exp}^{(\frac{{-\Delta H}_{vap}}{R} \left( \frac{1}{T}-\frac{1}{298} \right))}$ Eq. S4

where R (8.31 x 10-3 kJ/mol·K) is the ideal gas constant, T (K) is the average temperature across the deployment period, and 𝛥H_vap_ is the enthalpy of vaporization (kJ/mol).

The PRC sampling rate is then calculated for air R_sa,PRC_ :

$R_{sa,PRC}= - \frac{ln( \frac{N}{N_{0}})}{E}K_{sa(T)}V_{s}$ Eq. S5

Where N and N_0_ are the final and initial mass of PRCs, respectively, and E is the duration of sampler deployment. Sampling rates for target analytes are related to PRC sampling rates through the following equation^1^:

vapor-phase $R_{sa, analyte}=R_{sa, PRC}*\frac{\beta_{analyte}}{\beta_{PRC}}$ Eq. S6

b) $\log\beta=0.154{x logK}_{oa} -0.8$

(1) Huckins, J. N. P., J. D.; Booij, K. *Monitors of the organic chemicals in the environment*; Springer, 2006.

(2) Petty, J. D.; Huckins, J. N.; Martin, D. B.; Adornato, T. G. Use of semipermeable membrane devices (SPMDS) to determine bioavailable organochlorine pesticide residues in streams receiving irrigation drainwater. *Chemosphere* **1995**, *30* (10), 1891–1903. DOI: 10.1016/0045-6535(95)00070-O USGS Publications Warehouse.

(3) Lohmann, R. Critical Review of Low-Density Polyethylene’s Partitioning and Diffusion Coefficients for Trace Organic Contaminants and Implications for Its Use As a Passive Sampler. *Environmental Science & Technology* **2012**, *46* (2), 606–618. DOI: 10.1021/es202702y.

(4) Khairy, M. A.; Lohmann, R. Field Validation of Polyethylene Passive Air Samplers for Parent and Alkylated PAHs in Alexandria, Egypt. *Environmental Science & Technology* **2012**, *46* (7), 3990–3998. DOI: 10.1021/es300012u.

(5) Donald, C. E.; Anderson, K. A. Assessing soil-air partitioning of PAHs and PCBs with a new fugacity passive sampler. *Science of The Total Environment* **2017**, *596-597*, 293–302. DOI: <https://doi.org/10.1016/j.scitotenv.2017.03.095>.

(6) Minick, D. J.; Anderson, K. A. Diffusive flux of PAHs across sediment–water and water–air interfaces at urban superfund sites. *Environ. Toxicol. and Chem.* **2017**, *36* (9), 2281–2289. DOI: 10.1002/etc.3785.
